# Genome-Wide Association Study Reveals Candidate Genes Involved in Fruit Trait Variation in Persian Walnut (*Juglans regia* L.)

**DOI:** 10.3389/fpls.2020.607213

**Published:** 2021-01-08

**Authors:** Anthony Bernard, Julie Crabier, Armel S. L. Donkpegan, Annarita Marrano, Fabrice Lheureux, Elisabeth Dirlewanger

**Affiliations:** ^1^Univ. Bordeaux, INRAE, Biologie du Fruit et Pathologie, UMR 1332, Villenave d’Ornon, France; ^2^CTIFL, Centre Opérationnel de Lanxade, Prigonrieux, France; ^3^Department of Plant Sciences, University of California, Davis, Davis, CA, United States

**Keywords:** texture analyzer, gas chromatography, HPLC, X-ray CT, fruit traits, GWAS, *Juglans regia* L., walnut

## Abstract

Elucidating the genetic determinants of fruit quality traits in walnut is essential to breed new cultivars meeting the producers and consumers’ needs. We conducted a genome-wide association study (GWAS) using multi-locus models in a panel of 170 accessions of *Juglans regia* from the INRAE walnut germplasm collection, previously genotyped using the Axiom^TM^ *J. regia* 700K SNP array. We phenotyped the panel for 25 fruit traits related to morphometrics, shape, volume, weight, ease of cracking, and nutritional composition. We found more than 60 marker-trait associations (MTAs), including a highly significant SNP associated with nut face diameter, nut volume and kernel volume on chromosome 14, and 5 additional associations were detected for walnut weight. We proposed several candidate genes involved in nut characteristics, such as a gene coding for a beta-galactosidase linked to several size-related traits and known to be involved in fruit development in other species. We also confirmed associations on chromosomes 5 and 11 with nut suture strength, recently reported by the University of California, Davis. Our results enhance knowledge of the genetic control of important agronomic traits related to fruit quality in walnut, and pave the way for the development of molecular markers for future assisted selection.

## Introduction

Persian walnut (*Juglans regia* L.) is one of the oldest food sources known (Aradhya et al., 2006) and is subject to myths and legends since ancient times (Rondeau, 1997). It is a tree species with 2n = 2x = 32 chromosomes (Woodworth, 1930), growing in temperate region (McGranahan and Leslie, 2009). Walnut is an important tree crop in France after apple, with a worldwide in-shell walnut production led by China, California, and Iran^1^. If the ability of adapting to changing climatic conditions is among breeding priorities, larger fruit size, larger filling ratio, and easiness of cracking remain the main goals of most walnut breeding programs (Bernard et al., 2018b; Vahdati et al., 2019).

Effective management of genetic and phenotypic diversity within germplasm repositories is of valuable assistance to breeders. Typically, germplasm collections are first evaluated using morphological descriptors (Bernard et al., 2018b). Measurements of nut-related traits (e.g., shell thickness, nut length, nut diameter, etc.) in walnut were broadly conducted in Iran and Turkey using a caliper or a micrometer (Eskandari et al., 2005; Ghasemi et al., 2012; Ahandani et al., 2014; Khadivi-Khub et al., 2015; Mahmoodi et al., 2019). Similar studies were conducted in Europe (Zeneli et al., 2005; Poggetti et al., 2017), but the task is labor-intensive and not accurate. Careful consideration of the phenotyping method is of great importance since the heritability of a given trait may depend on the accuracy of the data (Furbank and Tester, 2011; Burghardt et al., 2017). Phenotyping techniques are evolving along with genotyping technologies, and X-ray computed tomography (CT) is one of the imaging techniques applied in food sciences to evaluate internal quality (Kotwaliwale et al., 2014), used notably for studying nut species such as almond, hazelnut, pecan (Harrison et al., 1993; Kim and Schatzki, 2001; Khosa and Pasero, 2014), and recently walnut (Bernard et al., 2020a).

Determination of genetic architecture of such traits has been the focus of several recent studies. A new era in walnut genetics started with the release of a high-density Axiom^TM^ *J. regia* 700K SNP genotyping array (Marrano et al., 2019a). By using this genomic tool, several association mappings studies have been possible. For instance, Arab et al. (2019) applied this SNP array to dissect the genetic architecture of nut-related traits, such as nut length, nut weight, shell thickness, shell texture, and kernel percentage. In addition, Marrano et al. (2019b) carried out a gene-mapping study to decipher the genetic control of yield, phenology, and kernel pellicle color. In addition, Sideli et al. (2020) investigated the genetic control of shell suture strength phenotyped using a texture analyzer, identifying many candidate genes. By combining acquisition of accurate phenotypic data using innovative techniques with the unique walnut germplasm collection from INRAE of Bordeaux, we studied the genetic control of 25 traits related to nut quality, and we propose several candidate genes involved in those traits. These achievements are the starting point for the selection and development of walnut cultivars with desirable and improved fruit quality features.

## Materials and Methods

### Plant Material and Phenotypic Data Acquisition

The INRAE walnut germplasm collection is publicly available and the GWAS panel is made of 170 unique *J. regia* accessions previously used to study phenological traits and lateral bearing genetic architecture (Bernard et al., 2020b). All the accessions are located in the Fruit Experimental Unit of the INRAE-Bordeaux research center, at Toulenne located 50 km south-west from Bordeaux, France. The accessions were classified into three groups according to the breeding level: Selection, Landrace and Modern varieties. All the accessions were collected from 1988 and 2000 from a collecting work in 23 countries.

Walnuts were phenotyped for 25 traits, important for producers or consumers but also for walnut industry. These traits can be classified into six groups as follows (Supplementary Table 1):

– Nut morphometric traits (nut length, nut face diameter, nut profile diameter, and nut surface area). These traits are particularly important for cultivar identification.
– Nut shape and texture traits (nut shape VA3D, nut Feret shape VA3D, nut sphericity, and shell rugosity), decisive for the attractiveness to the consumers.
– Volume traits (nut, shell, kernel, and empty space volumes, and kernel filling ratio), that determine the breaking yield.
– Weight traits (nut weight, x3 extreme groups, kernel weight, and breaking yield).
– Shell cracking related traits (nut face strength, nut suture strength, and shell thickness), crucial for walnut industry.
– Nutritional components (saturated fatty acids, polyunsaturated fatty acids, monounsaturated fatty acids, tocopherols, and vitamin E activity).

In-shell walnut sampling (*n* = 100 nuts/accession) was performed during harvest seasons in September 2017, 2018, 2019 for each accession. Walnuts were dried for 2 days at 25°C using a food dryer. They were stored in a cold room set to 2°C. We phenotyped 25 traits.

Nut morphometric traits were evaluated on nuts collected in 2017, nut length, nut face diameter and nut profile diameter were measured using an electronic caliper (accuracy 10^–2^ mm) on the sample of 100 nuts/accession. On nuts collected in 2018, all traits were measured using an X-ray CT method on a subsample of 50 nuts randomly selected from the 100 nuts original sample collected. X-ray CT scans and analyses were performed as described by Bernard et al. (2020a). Nut shape and texture traits (nut shape VA3D, nut Feret shape VA3D, nut sphericity, and shell rugosity) were measured using the X-ray CT method on nuts collected in 2018. Volume traits were measured using the X-ray CT method on nuts collected in 2018. Weight traits were measured on nuts collected in 2017, 2018, and 2019 using an electronic scale (accuracy 10^–1^ mm). A subsample of 50 nuts from the 100 nuts sampled every year was randomly selected to obtain the weight of 50 kernels and the breaking yield. The French walnut industry considers seven size groups depending on the diameter: < 28 mm, 28–30 mm, 30–32 mm, 32–34 mm, 34–36 mm, 36–38 mm, > 38 mm. The trait “x3 extreme groups” is the ratio of the number of walnuts in the three extreme groups (34–36 mm, 36–38 mm, and > 38 mm) to the total number of walnuts in all groups. For shell cracking related traits a subsample of 50 randomly selected nuts was halved to determine the force needed to crack the nut (initial rupture) on the face (25 nuts) and on the suture (25 nuts) for 2017, 2018, and 2019 samples using a texture analyzer (TA-PLUS model, TA1 Texture Analyzer series, from Lloyd Materials Testing^TM^, Ametek®). The following parameters were retained: compression speed of 75 mm/min and detection of the initial break when the force drops abruptly by 80%. For the 2018 harvest, the shell thickness was obtained using the X-ray CT method. Nutritional components were measured on nuts collected in 2018 using the following methods: fatty acids were extracted by 3/2 (v/v) hexane/isopropanol mixture, miscella washed with 0.8% potassium chloride, vacuum evaporated with a Buchi^®^ rotary evaporator. Fatty acid methyl esters were analyzed using gas chromatography according to NF EN ISO 12966-2 and 12966-4 standards, whereas tocopherols/tocotrienols were processed using high-performance liquid chromatography according to the NF EN ISO 9936 standard. Quantifications were performed at ITERG laboratory (Canéjan, France).

### Data Analysis, SNP Genotyping, Population Structure, and Kinship Analyzes

Data management and visualization were performed using “R” software with the package “tidyverse” (Wickham, 2017). The Pearson correlation matrix was performed using the package “corrplot” (Wei and Simko, 2017). The Principal Component Analysis (PCA) was performed using the package “FactoMineR” (Lê et al., 2008). The means of genotypic effects were obtained for each accession by adjusting for year effect using the Best Linear Unbiased Predictions (BLUPs), considering the following mixed linear model:

$$P_{i\,k}=\mu +Y_{i}+g_{k}+e_{i\,k}$$

where *P*_*ik*_ refers to the observed phenotype of the *k*th accession in the *i*th year; μ is the mean value of the trait; *Y*_*i*_ is the fixed effect of the *i*th year, *g*_*k*_ is the random effect of the *k* genotype; and *e*_*ik*_ is the residuals of the model. The BLUPs were performed using the package “lme4” (Bates et al., 2015). The broad-sense heritability of the traits phenotyped during at least 2 years was estimated using the variance components obtained by the previous mixed linear model:

$$H^{2}=\sigma_{G}^{2}/\sigma_{P}^{2}$$

where σ^2^*_*G*_* is the genotypic effect variance and σ^2^*_*P*_* is the phenotypic variance.

The accessions were genotyped using the Axiom^TM^ *J. regia* 700K SNP array containing 609,658 SNPs uniformly distributed over the 16 *J. regia* chromosomes (Marrano et al., 2019a). These SNPs were then filtered through several criteria described previously (Bernard et al., 2020b). Finally, 364,275 robust SNPs were retained for the GWAS.

The population structure was investigated using the “fastSTRUCTURE” software and the most likely K was determined using the ΔK method (Bernard et al., 2020b). The identity-by-descent (IBD) proportions between all pairwise comparisons are already described in Bernard et al. (2020b).

### Genome-Wide Association Analysis, LD Blocks, and Search of Annotations

GWAS was carried out using the R package “GAPIT” (Lipka et al., 2012). Two multi-locus models were tested using the BLUPs as phenotypic data: the Multi-Locus Mixed Model (MLMM) (Segura et al., 2012) and the Fixed and random model Circulating Probability Unification method (FarmCPU) (Liu et al., 2016), as already described (Bernard et al., 2020b). Familiar relatedness was accounted for using a kinship matrix estimated with the VanRaden algorithm implemented in GAPIT. In order to correct for population structure, the best number of principal components to include in our models was selected using the “model.selection” function implemented in GAPIT according to the Bayesian Information Criterion (BIC). Significant marker-trait associations (MTAs) were determined using both 1 and 5% Bonferroni correction, and previous knowledge based on literature for cracking related traits. The percentage explained variance *R*^2^ was corrected for genome-wide background.

Each physical position of the identified MTAs was investigated to explore the extension of the surrounding linkage disequilibrium (LD) blocks using “solid spine of LD” method implemented in HaploView v4.2 software (Barrett et al., 2005). We searched the defined LD blocks for candidate genes using the walnut nuclear gene annotation and mapped into the new chromosome-scale reference “Chandler” genome v2.0 (Marrano et al., 2020)^2^.

## Results

### Phenotypic Data

A large variability was observed for all traits within the INRAE walnut germplasm collection (Table 1). Considering the range of the nut length in 2017, the values vary from ∼26 to 53 mm according to the accessions. We observed higher variation for nut volume in 2018 (from ∼10,400 to 43,000 mm^3^), nut weight in 2017 (from ∼522 to 2,278 g), and for nut suture strength in 2019 (from ∼101 to 777 N). On the contrary, there was low variation for nut sphericity (from 0.84 to 0.93) and the proportion of saturated fatty acids (from 8.60 to 11.29%). The distribution of the 25 studied traits is shown in Supplementary Figure 1.

**TABLE 1:** Descriptive statistics and broad-sense heritability values of the 25 traits studied in the walnut GWAS analysis.

Trait	Unit^a^	Year	Mean ± SD^b^	Range	*H*^2^
**Nut morphometrics related traits**					
Nut length	mm	2017	37.53 ± 4.20	25.99–52.69	0.91
		2018	38.39 ± 3.91	28.57–51.43	
Nut face diameter	mm	2017	31.54 ± 2.99	23.01–40.55	0.79
		2018	32.27 ± 2.55	25.99–40.75	
Nut profile diameter	mm	2017	32.25 ± 3.41	24.10–42.87	0.83
		2018	33.29 ± 2.88	27.06–42.84	
Nut surface area	mm^2^	2018	4,019.54 ± 701.70	2,622.59–7,093.53	–
**Nut shape and texture related traits**					
Nut shape VA3D	–	2018	1.47 ± 0.10	1.24–1.69	–
Nut feret shape 3D	–	2018	1.25 ± 0.08	1.12–1.48	–
Nut sphericity	–	2018	0.88 ± 0.02	0.84–0.93	–
Shell rugosity	–	2018	1.14 ± 0.03	1.08–1.19	–
**Volume related traits**					
Nut volume	mm^3^	2018	19,400.02 ± 4,889.76	10,382.05–42,813.08	–
Shell volume	mm^3^	2018	4,076.78 ± 877.94	2,390.66–9,051.88	–
Kernel volume	mm^3^	2018	5,723.89 ± 1,152.14	3,408.85–9,548.93	–
Empty space volume	mm^3^	2018	9,599.35 ± 3,226.42	4,536.51–24,212.21	–
Kernel filling ratio	%	2018	30.00 ± 3.38	20.70–37.40	–
**Weight related traits**					
Nut weight	g	2017	1,109.64 ± 262.52	521.74–2,278.20	0.86
		2018	1,192.80 ± 255.37	624.40–2,251.40	
		2019	1,152.86 ± 263.43	539.98–2,288.86	
X3 extreme groups	%	2017	30.97 ± 35.88	0.00–100.00	0.82
		2018	46.51 ± 35.24	0.00–100.00	
		2019	33.65 ± 35.57	0.00–100.00	
Kernel weight	g	2017	249.27 ± 61.40	113.72–416.40	0.77
		2018	276.12 ± 56.51	138.50–440.70	
		2019	260.18 ± 56.84	146.73–428.03	
Breaking yield	%	2017	44.86 ± 5.23	30.03–65.85	0.80
		2018	45.45 ± 5.11	30.80–59.40	
		2019	46.03 ± 5.09	30.47–60.54	
**Cracking related traits**					
Nut face strength	N	2017	435.18 ± 106.69	205.72–763.34	0.71
		2018	424.24 ± 119.58	182.80–893.70	
		2019	409.53 ± 105.89	172.41–863.61	
Nut suture strength	N	2017	281.06 ± 102.16	87.49–614.37	0.72
		2018	250.31 ± 88.59	74.40–657.20	
		2019	308.62 ± 104.20	101.35–776.97	
Shell thickness	mm	2018	1.03 ± 0.11	0.73–1.49	–
**Nutritional components**					
Saturated fatty acids	%	2018	9.90 ± 0.57	8.60–11.29	–
Polyunsaturated fatty acids	%	2018	72.36 ± 2.70	63.91–78.13	–
Monounsaturated fatty acids	%	2018	17.60 ± 2.73	11.77–25.33	–
Tocopherols	mg/kg	2018	371.07 ± 110.19	40.00–646.00	–
Vitamin E activity	mg alpha-TE/100 g	2018	4.27 ± 1.62	0.20–8.30	–

*^*a*^Units abbreviations: mm, millimeter; mm^2^, square millimeter; mm^3^, cubic millimeter; %, percentage; g, gram; N, Newton; alpha-TE, alpha-tocopherol equivalent. ^*b*^SD, standard deviation.*

**FIGURE 1:**
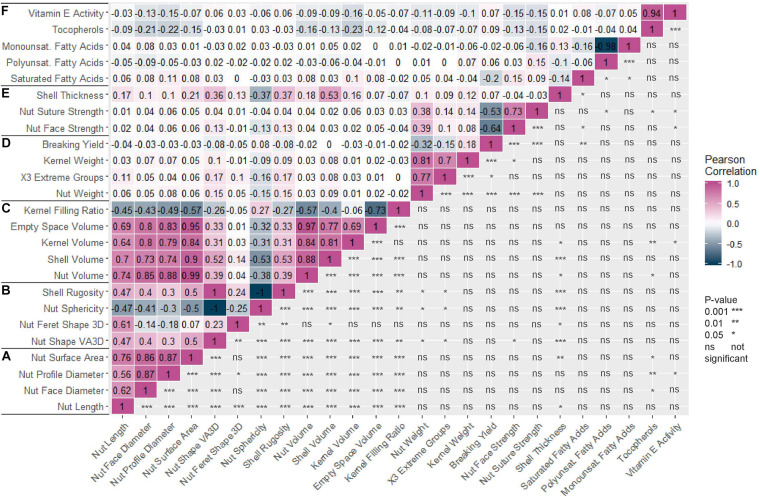
Correlation matrix of the 25 traits studied in the walnut GWAS analysis. **(A)** Nut morphometric traits. **(B)** Nut shape and texture traits. **(C)** Volume traits. **(D)** Weight traits. **(E)** Cracking traits. **(F)** Nutritional components. Pearson correlation coefficient was used and *p*-value is indicated as follows: 0.001 (***), 0.01 (**), 0.05 (*), not significant (ns).

The Pearson correlation matrix (Figure 1) indicated strong positive correlations between all nut morphometrics related traits (group a) and volume related traits (group c). For instance, nut length was positively correlated with nut face diameter (0.62), nut volume (0.74), and kernel volume (0.64). Nut weight was also positively correlated with kernel weight (0.81). Nut suture and nut face strengths were positively correlated (0.73) but both were negatively correlated with breaking yield (−0.53 and −0.64 respectively). Nut sphericity was negatively correlated with shell rugosity and nut shape “VA3D” (−1 for both). Kernel filling ratio was negatively correlated with nut volume (−0.57). Finally, breaking yield, which is the ratio between the weight of the kernel and the nut, was not correlated with kernel filling ratio, which is the same ratio but based on volumes.

The broad-sense heritability values estimated for the nine traits measured in at least two years showed differences (Table 1). Nut morphometrics related traits had high *H*^2^-values, ranging from 0.79 to 0.91, as those related to the weight (from 0.77 to 0.86). However, nut face strength and suture strength had smaller *H*^2^-values, 0.71 and 0.72, respectively, indicating a more complex genetic architecture.

### BLUPs and Cofounding Factors

**FIGURE 2:**
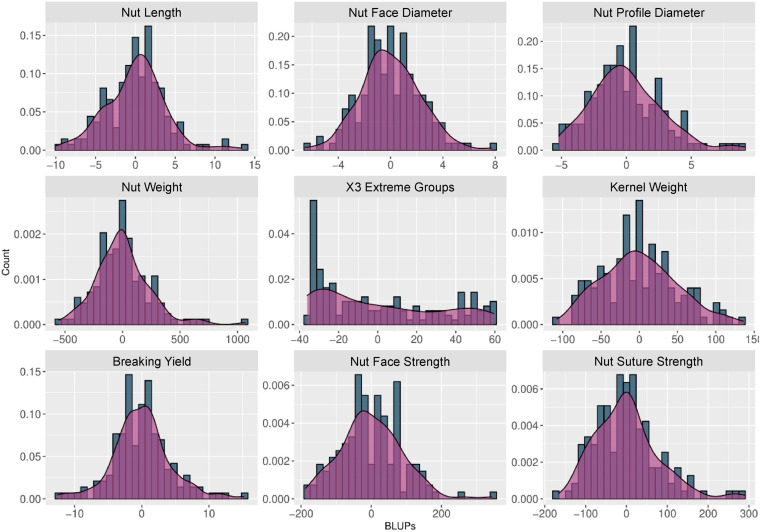
Histograms and density plots of the BLUPs used for the walnut GWAS analysis. The BLUPs were calculated for the 9 traits observed during at least 2 years using a linear mixed model, considering the genotypic mean as a random effect and the year as a fixed effect.

BLUPs were calculated for the nine traits with at least 2 years of phenotypic data using a linear mixed model that included the genotypic mean as a random effect and the year as a fixed effect. The density plots (Figure 2) showed a normal distribution for all traits, except for “x3 extreme groups” trait.

**FIGURE 3:**
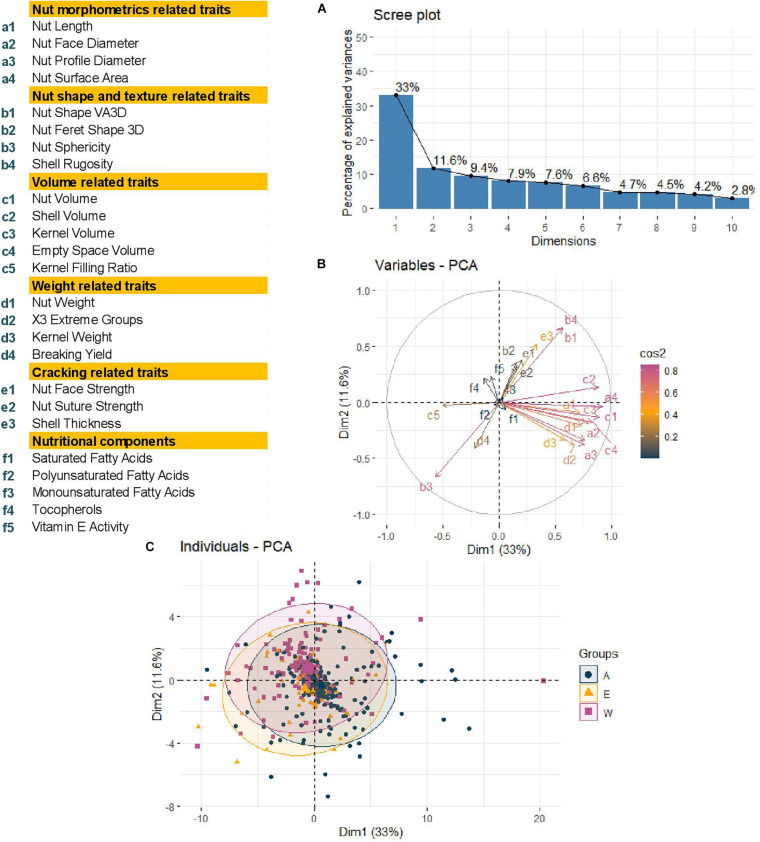
Principal Component Analysis performed using phenotypic raw dataset of the walnut GWAS analysis. **(A)** Scree plot indicating the percentage of explained variances of the first 10 dimensions. **(B)** Variables circular plot indicating the cos^2^ of the 25 traits for the two first two dimensions. **(C)** Individual scatterplot indicating the accessions colored by results of structure analysis previously performed. Groups: A, admixed, E, Eastern Europe and Asia, W, Western Europe and America.

Using a BIC, we defined that the best number of principal components to include in the models for accounting for structure was zero, as previously found for phenological traits (Bernard et al., 2020b). We also determined the best K as *K* = 2 with a group consisting of the accessions coming from Western Europe and America and the second with the accessions from Eastern Europe and Asia (Bernard et al., 2020b). As a result, we decided to perform a Principal Component Analysis (PCA) using the 25 traits and structure-based individual clustering (Figure 3). The first two dimensions of the PCA explained 44.6% of the total variance (Figure 3A). Along the first dimension, individuals grouped according to the morphometrics, volume and weight-related traits, whereas the dimension 2 separated the walnut accessions based on shape, texture, and cracking related traits (Figure 3B). We could also notice that all groups (Western Europe and America “W,” Eastern Europe and Asia “E,” and the admixed “A”) contained accessions with large or small-sized nuts (Figure 3C), since the 95% confidence ellipses intersected and blended together. This means that the structure of our germplasm did not influence the traits, supporting the best number of PCs to include of zero.

### MTAs for Fruit Traits

**FIGURE 4:**
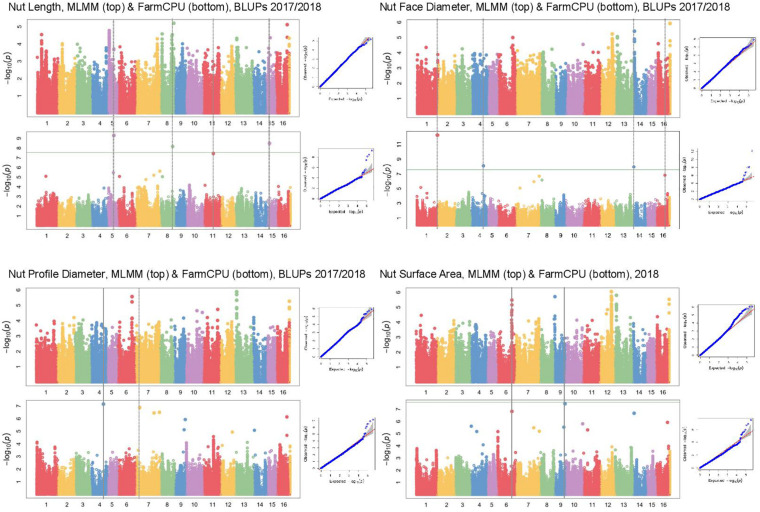
Manhattan plots of nut morphometrics related traits in the walnut GWAS analysis. For each trait, MLMM Manhattan plot and Q-Q plot are shown at the top, and FarmCPU Manhattan plot and Q-Q plot are shown at the bottom. Horizontal green line corresponds to 1% Bonferroni threshold automatically implemented in GAPIT.

**FIGURE 5:**
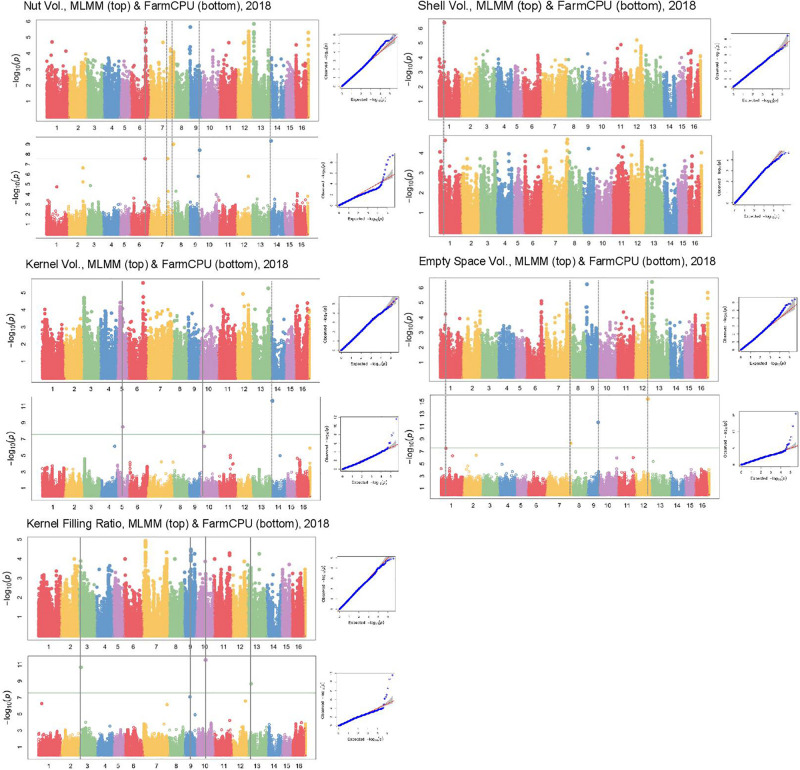
Manhattan plots of volume related traits in the walnut GWAS analysis. For each trait, MLMM Manhattan plot and Q-Q plot are shown at the top, and FarmCPU Manhattan plot and Q-Q plot are shown at the bottom. Horizontal green line corresponds to 1% Bonferroni threshold automatically implemented in GAPIT.

**FIGURE 6:**
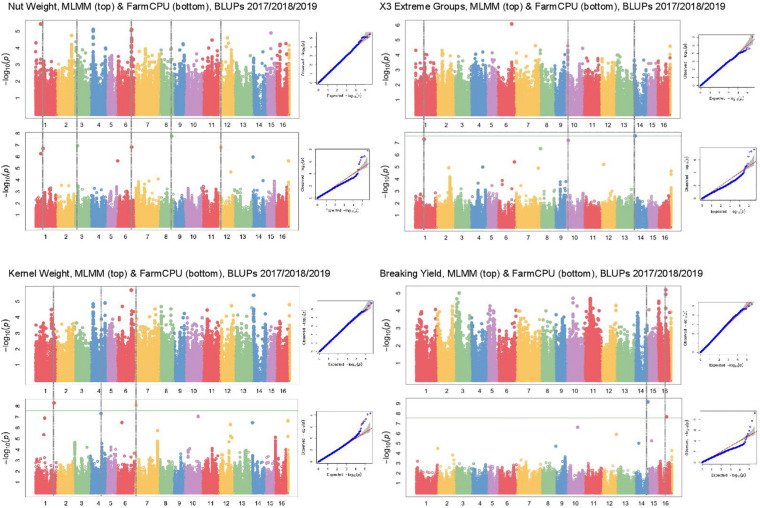
Manhattan plots of weight related traits in the walnut GWAS analysis. For each trait, MLMM Manhattan plot and Q-Q plot are shown at the top, and FarmCPU Manhattan plot and Q-Q plot are shown at the bottom. Horizontal green line corresponds to 1% Bonferroni threshold automatically implemented in GAPIT.

In Manhattan plots, the –log_10_ of the *p*-value were equal to 7.56, giving 37 MTAs using 1% Bonferroni threshold automatically implemented in GAPIT. By using 5% Bonferroni threshold, the –log_10_ of the *p*-value were equal to 6.86, giving 58 significant MTAs in total (Figures 4–6). We found on average 3.2 MTAs per trait. For instance, we found MTAs on chromosomes (Chr) 5, 8, 11, and 15 for nut length (Figure 4), on Chr6, 7, 9, 14 for nut volume (Figure 5) and on Chr1, 3, 6, 8, and 12 for nut weight (Figure 6). Most of the significant MTAs were obtained using the FarmCPU model, whereas the MLMM approach identified two SNPs associated with both tocopherols content and vitamin E activity (Supplementary Figure 3). However, the Manhattan plots indicated strong similarities between both MLMM and FarmCPU models for all traits, even if the signals obtained with the MLMM were below the significant thresholds. We found no significant associations for 6 traits: nut shape VA3D, nut sphericity, nut rugosity, shell volume, saturated and polyunsaturated fatty acids content (Supplementary Figure 3).

For some of the correlated traits, we detected MTAs which were very close or even at the same physical position, that we called loci of interest (LOI) (Table 2). For instance, MTA AX-171125096 (Chr4, 26,041,265 bp) was associated with both nut face diameter (*R*^2^ = 19.80) and nut profile diameter (*R*^2^ = 16.50), even though the single traits exhibited additional associations signals on other chromosomes (e.g., on Chr1 for the nut face diameter and on Chr7 for the nut profile diameter). This locus was referred as LOI2 in Table 2. Moreover, MTA AX-170690867 (Chr14, 1,248,953 bp) was associated with nut face diameter, nut volume, and kernel volume, and was only 119 bp apart from MTA AX-171170293 associated with “x3 extreme groups” trait. This locus included these two MTAs, was referred to LOI3 (Table 2). Similarly, the MTA AX-170984383 on Chr9 for nut surface area was also found for nut volume and empty space volume (referred as LOI4).

**TABLE 2:** List of the MTAs identified for the 25 traits studied in the walnut GWAS analysis with their position, significance and effect.

MTAs	Chr^a^	Physical position	Significance/Model^b^	i^2c^	Alleles/Effect^d^	LOI^e^ for several traits
**Nut morphometrics related traits**						
**Nut length**						
AX-170748449	5	13,134,800	5.02E-10/FarmCPU**	14.45	T,C/2.87	LOI1
AX-170808025	15	4,444,194	3.23E-09/FarmCPU**	15.40	T,G/1.47	
AX-171077810	8	25,778,592	6.88E-09/FarmCPU**	< 0.10	A,G/1.23	
AX-171191763	11	20,831,595	3.66E-08/FarmCPU*	14.50	C,T/2.44	
**Nut face diameter**						
AX-171110506	1	44,683,085	5.17E-13/FarmCPU**	5.88	A,G/0.90	
AX-171125096	4	26,041,265	7.98E-09/FarmCPU**	19.80	T,C/-1.41	LOI2
AX-170690867	14	1,248,953	1.11E-08/FarmCPU**	26.36	G,T/0.74	LOI3
AX-170799076	16	19,169,530	1.47E-07/FarmCPU*	3.22	T,G/0.67	
**nut profile diameter**						
AX-171125096	4	26,041,265	7.10E-08/FarmCPU*	16.50	T,C/-1.48	LOI2
AX-171059229	7	7,823,815	1.33E-07/FarmCPU*	< 0.10	T,C/0.72	
**Nut surface area**						
AX-170984383	9	22,804,956	3.58E-08/FarmCPU*	16.51	A,C/250.39	LOI4
AX-171001086	6	30,535,497	1.50E-07/FarmCPU*	16.11	T,G/-327.60	LOI5
**Nut shape and texture related traits**						
**Nut shape VA3D**	No association	–	–	–	–	
**Nut feret shape 3D**						
AX-170668694	13	4,932,826	2.20E-11/FarmCPU**	13.16	C,T/-0.07	
**Nut sphericity**	No association	–	–	–	–	
**Nut rugosity**	No association	–	–	–	–	
**Volume related traits**						
**Nut volume**						
AX-170690867	14	1,248,953	4.66E-10/FarmCPU**	17.33	G,T/2,101.08	LOI3
AX-171514816	7	50,526,399	1.00E-09/FarmCPU**	16.39	A,T/2,018.71	LOI6
AX-170984383	9	22,804,956	3.95E-09/FarmCPU**	16.38	A,C/1,783.58	LOI4
AX-170810656	7	38,570,409	2.76E-08/FarmCPU*	<0.10	T,C/-1,574.13	
AX-171001086	6	30,535,497	2.76E-08/FarmCPU*	17.09	T,G/-2,228.97	LOI5
AX-170719786	2	31,237,408	2.35E-07/FarmCPU*	<0.10	G,A/-1,760.19	
**Shell volume**	No association	**–**	**–**	**–**	**–**	
**Kernel volume**						
AX-170690867	14	1,248,953	2.13E-12/FarmCPU**	24.97	G,T/521.01	LOI3
AX-170640054	5	10,793,840	3.24E-09/FarmCPU**	21.02	G,A/-586.65	
AX-170940799	10	6,432,676	1.41E-08/FarmCPU**	19.54	C,A/-594.42	
**Empty space volume**						
AX-170593629	12	24,730,815	3.45E-16/FarmCPU**	15.03	A,C/-1,717.71	
AX-170984383	9	22,804,956	2.01E-12/FarmCPU**	17.01	A,C/1,462.60	LOI4
AX-171514726	7	50,189,503	4.52E-09/FarmCPU**	17.06	C,G/1,324.30	LOI6
AX-171017658	1	10,234,077	3.27E-08/FarmCPU**	16.07	C,A/-2,014.48	
**Kernel filling ratio**						
AX-171048523	10	19,688,911	2.75E-12/FarmCPU**	14.98	C,T/0.02	
AX-171583500	3	3,603,319	2.12E-11/FarmCPU**	20.44	T,A/0.01	
AX-171540343	13	6,539,102	2.16E-09/FarmCPU**	0.13	A,G/-0.01	
AX-171117250	9	12,230,952	8.33E-08/FarmCPU*	7.17	T,C/0.01	
**Weight related traits**						
**Nut weight**						
AX-171175345	8	26,618,252	1.72E-08/FarmCPU**	0.59	C,T/-176.41	
AX-170573680	3	7,355,283	1.20E-07/FarmCPU*	2.10	G,T/-64.13	
AX-171083810	6	31,463,066	1.54E-07/FarmCPU*	4.23	T,G/-104.12	
AX-170973887	12	3,304,218	1.62E-07/FarmCPU*	17.13	A,G/67.16	
AX-170605550	1	15,926,539	2.02E-07/FarmCPU*	3.47	A,G/50.24	
**X3 extreme groups**						
AX-171170293	14	1,248,839	2.68E-08/FarmCPU**	21.7	A,G/-9.83	LOI3
AX-170834489	1	19,207,870	5.05E-08/FarmCPU*	7.02	T,C/8.82	
AX-170723157	10	4,802,938	7.00E-08/FarmCPU*	5.01	T,C/-12.56	
**Kernel weight**						
AX-171207844	1	39,963,556	5.17E-09/FarmCPU**	11.50	G,T/15.27	
AX-171547969	7	3,283,684	8.25E-09/FarmCPU**	4.66	T,A/-11.89	LOI7
AX-170806411	4	23,487,069	4.85E-08/FarmCPU*	10.54	C,A/28.31	
**Breaking yield**						
AX-171005810	14	26,821,528	6.80E-10/FarmCPU**	0.72	C,A/2.55	LOI8
AX-170746651	16	17,458,649	2.11E-08/FarmCPU**	27.23	A,G/-2,09	
**Cracking related traits**						
**Nut face strength**						
AX-170722428	11	13,511,848	9.10E-06/FarmCPU	16.13	G,A/-53.53	LOI9
AX-170865366	2	32,096,896	2.49E-06/MLMM	11.24	G,A/NA	LOI10
**Nut suture strength**						
AX-170748526	5	13,024,522	2.82E-07/MLMM	12.26	C,A/NA	LOI1
AX-170908256	11	12,909,083	6,72E-05/FarmCPU	14.75	G,A/-49.90	LOI9
**Shell thickness**						
AX-170865411	2	32,128,087	1.46E-07/MLMM	13.69	A,C/NA	LOI10
**Nutritional components**						
**Saturated fatty acids**	No association	**–**	**–**	**–**	**–**	
**Polyunsaturated fatty acids**	No association	**–**	**–**	**–**	**–**	
**Monounsaturated fatty acids**						
AX-170701328	14	26,730,713	2.73E-12/FarmCPU**	17.73	C,T/−1.43	LOI8
AX-171494826	9	21,100,618	3.81E-10/FarmCPU**	13.58	A,T/−2.19	
AX-170973656	12	3,155,205	1.42E-08/FarmCPU**	13.35	T,C/0.90	
AX-170996807	14	21,159,561	5.63E-08/FarmCPU*	< 0.10	A,G/0.86	
AX-171536319	7	47,712,322	7.00E-08/FarmCPU*	14.27	G,C/−1.72	
**Tocopherols**						
AX-171134134	10	34,010,861	7.21E-13/MLMM**	14.03	C,T/NA	LOI11
AX-170893393	9	15,280,292	6.22E-09/MLMM**	7.13	C,T/NA	LOI12
AX-170775823	13	23,062,305	5.98E-15/FarmCPU**	12.60	G,T/95.00	
AX-171016396	17^f^	–	8.10E-11/FarmCPU**	4.55	C,A/−40.48	
AX-170615496	8	29,149,403	1.02E-09/FarmCPU**	18.16	A,G/81.85	
AX-171592220	5	16,978,410	9.09E-09/FarmCPU**	< 0.10	C,G/−41.90	
AX-170909834	7	3,266,820	3.89E-08/FarmCPU*	11.70	T,C/73.88	LOI7
AX-171595297	14	7,508,539	4.40E-08/FarmCPU*	11.80	A,T/65.54	
**Vitamin E activity**						
AX-171134134	10	34,010,861	7.93E-13/MLMM**	13.78	C,T/NA	LOI11
AX-1708 93393	9	15,280,292	2.16E-08/MLMM**	5.32	C,T/NA	LOI12

**Association significant according to Bonferroni correction at 0.01 using the mentioned model. *Association significant according to Bonferroni correction at 0.05 using the mentioned model. No asterisk indicates that the association is not significant but of interest regarding Manhattan plot of the mentioned model and previous knowledge. ^*a*^Chr, Chromosome. ^*b*^The significance value indicated is the unadjusted p-value. ^*c*^R^2^, percentage explained variance corrected for genome-wide background. ^*d*^The allelic effect is the difference in mean of measured trait between genotypes with one or other allele. The sign (±) is with respect to the major allele that is second-mentioned. ^*e*^LOI, locus of interest associated to several traits referred to a unique MTA or to several MTAs with close physical position. ^*f*^Chr 17, virtual chromosome corresponding to all unmapped SNPs.

For the cracking-related traits, no signals passed the significant thresholds. However, we observed interesting peaks on Chr2 and 11 for nut face strength, on Chr5 and 11 for nut suture strength, and on Chr2 for shell thickness, which was very close to the one found with the nut face strength (Table 2). Since significant associations on Chr5 and 11 for the suture strength have been previously reported, we decided to explore and discuss further these signals.

### LD Blocks and Candidate Genes

Using the “solid spine of LD” method, we found differences in LD level around the MTAs. The LOI3, including the MTA AX-170690867 (Chr14, 1,248,953 bp) associated with nut face diameter, nut volume, and kernel volume, and the MTA AX-171170293 (Chr14, 1,248,839 bp) linked with “x3 extreme groups,” belong to a very short LD block of 814 bp (Table 3). On the contrary, the LD block around the MTA AX-171514816 on Chr7, associated with nut volume, extends for 139,342 bp. We also found several loci in complete linkage equilibrium, such as the MTA AX-171001086 (Chr6, 30,535,497 bp), associated with the nut surface area, which did not belong to any block.

**TABLE 3:** List of the MTAs for 25 walnut traits with the candidate genes located in the corresponding LD block.

MTAs	Chr^a^	Physical position^b^	LD block interval^b,c^	Gene ID v2.0 genome	Gene ID v1.0 genome	Gene interval^b^	Functional annotation^d^	LOI ^e^ forn several traits ^e^
**Nut morphometrics related traits**								
**Nut length**								
AX-170748449	5	13,134,800	13,128,354–13,151,244	No gene	–	–	–	
AX-170808025	15	4,444,194	4,439,011–4,444,527	No gene	–	–	–	
AX-171077810	8	25,778,592	25,778,592–25,778,723	Jr08_18480, Jr08_18490, Jr08_18500	108979693	25,774,862–25,778,977	**Receptor-like cytosolic serine/threonine-protein kinase RBK1**	
AX-171191763	11	20,831,595	20,831,267–20,865,013	Jr11_12080	109017990	20,831,874–20,834,965	Death-associated protein kinase 1	
**Nut face diameter**								
AX-171110506	1	44,683,085	44,682,326–44,689,338	Jr01_34160	108982082	44,678,366–44,683,047	Transcriptional regulator STERILE APETALA	
AX-171125096	4	26,041,265	26,031,692–26,041,265	No gene	–	–	–	
AX-170690867	14	1,248,953	1,248,139–1,248,953	Jr14_01880	109012316	1,248,515–1,262,435	**Beta-galactosidase**	LOIA3
AX-170799076	16	19,169,530	19,167,864–19,170,797	No gene	–	–	–	
**Nut profile diameter**								
AX-171125096	4	26,041,265	26,031,692–26,041,265	No gene	–	–	–	
AX-171059229	7	7,823,815	7,819,426–7,829,301	No gene	–	–	–	
**Nut surface area**								
AX-170984383	9	22,804,956	22,785,041–22,811,656	Jr09_14670	108992222	22,786,325–22,787,008	Calcium-binding protein PBP1	LOI4
				Jr09_14680	108993282	22,793,835–22,797,249	G-type lectin S-receptor-like serine/threonine-protein kinase At4g27290	
				Jr09_14690	108993306	22,807,448–22,811,241	NAD(P)H dehydrogenase (quinone) FQR1	
AX-171001086	6	30,535,497	No block	Jr06_14940	108998609	30,533,899–30,541,448	**L-Ala-D/L-amino acid epimerase**	LOI5
**Nut shape and texture related traits**								
**Nut feret shape 3D**								
AX-170668694	13	4,932,826	4,927,503–4,935,968	Jr13_06810, Jr13_06820	109010712	4,929,379–4,935,469	**Probable E3 ubiquitin-protein ligase ARI1 isoform X1**	
**Volume related traits**								
**Nut volume**								
AX-170690867	14	1,248,953	1,248,139–1,248,953	Jr14_01880	109012316	1,248,515–1,262,435	**Beta-galactosidase**	LOI3
AX-171514816	7	50,526,399	50,423,525–50,562,867	Jr07_36670	108986372	50,425,702–50,428,580	Receptor-like cytoplasmic kinase 176	
				Jr07_36680, Jr07_36690	108986373	50,429,399–50,432,932	Probable dual-specificity RNA methyltransferase RlmN	
				Jr07_36700	108986346	50,434,181–50,439,305	Serine/arginine-rich SC35-like splicing factor SCL30	
				Jr07_36710	108986347	50,440,280–50,442,223	Elicitor-responsive protein 1	
				Jr07_36740	108986342	50,446,655–50,461,694	AP3-complex subunit beta-A	
				Jr07_36760, Jr07_36770	108986361	50,493,634–50,497,443	Protein INVOLVED IN *DE NOVO* 2	
				Jr07_36780	108986296	50,500,733–50,502,965	Expansin-A4	
				Jr07_36790	108986359	50,520,505–50,526,244	Telomere repeat-binding protein 4	
				Jr07_36800	108986374	50,540,228–50,542,459	Scarecrow-like protein 14	
				Jr07_36810, Jr07_36820	108986306	50,544,143–50,545,923	Ribosomal RNA large subunit methyltransferase E	
				Jr07_36830	108986354	50,547,343–50,550,514	Protein FIZZY-RELATED 3	
				Jr07_36840	108986353	50,551,233–50,557,760	Actin-related protein 5	
AX-170984383	9	22,804,956	22,785,041–22,811,656	Jr09_14670	108992222	22,786,325–22,787,008	Calcium-binding protein PBP1	LOI4
				Jr09_14680	108993282	22,793,835–22,797,249	G-type lectin S-receptor-like serine/threonine-protein kinase At4g27290	
				Jr09_14690	108993306	22,807,448–22,811,241	NAD(P)H dehydrogenase (quinone) FQR1	
AX-170810656	7	38,570,409	38,568,209–38,618,466	Jr07_22410	108990569	38,582,842–38,586,425	Oxidation resistance protein 1	
				Jr07_22420, Jr07_22430	109019525	38,587,432–38,601,941	Pre-mRNA-processing factor 19	
				Jr07_22440	109019524	38,617,705–38,622,029	Zinc finger protein CO3	
AX-171001086	6	30,535,497	No block	Jr06_14940	108998609	30,533,899–30,541,448	**L-Ala-D/L-amino acid epimerase**	LOI5
AX-170719786	2	31,237,408	31,218,056–31,252,334	Jr02_18130	109014448	31,222,938–31,226,154	RNA-binding protein NOB1	
				Jr02_18140	109014447, 109004211	31,227,157–31,232,427	Probable LRR receptor-like serine/threonine-protein kinase At1g63430	
				Jr02_18150	109014446	31,244,247–31,248,754	Protein O-glucosyltransferase 2	
**Kernel volume**								
AX-170690867	14	1,248,953	1,248,139–1,248,953	Jr14_01880	109012316	1,248,515–1,262,435	**Beta-galactosidase**	LOI3
AX-170640054	5	10,793,840	10,792,541–10,794,446	No gene	–	–	–	
AX-170940799	10	6,432,676	6,429,804–6,441,312	Jr10_08990	108996735	6,431,741–6,432,166	Tubulin beta-1 chain	
**Empty space volume**								
AX-170593629	12	24,730,815	24,688,102–24,738,026	Jr12_15420	109005084	24,689,734–24,690,792	Early light-induced protein 1, chloroplastic	
				Jr12_15430, Jr12_15440, Jr12_15450	109005081	24,691,848–24,699,404	Dymeclin	
				Jr12_15460	109005079	24,705,762–24,707,383	Heat stress transcription factor A-6b	
				Jr12_15470	109005080	24,709,136–24,712,428	1,2-dihydroxy-3-keto-5-methylthiopentene dioxygenase 2	
				Jr12_15480	109005077	24,723,413–24,725,999	1,2-dihydroxy-3-keto-5-methylthiopentene dioxygenase 1	
				Jr12_15490, Jr12_15500	109005076	24,728,030–24,733,224	**protein TIFY 4B**	
AX-170984383	9	22,804,956	22,785,041–22,811,656	Jr09_14670	108992222	22,786,325–22,787,008	Calcium-binding protein PBP1	LOI4
				Jr09_14680	108993282	22,793,835–22,797,249	G-type lectin S-receptor-like serine/threonine-protein kinase At4g27290	
				Jr09_14690	108993306	22,807,448–22,811,241	NAD(P)H dehydrogenase (quinone) FQR1	
AX-171514726	7	50,189,503	50,141,099–50,199,957	Jr07_36190	108986377	50,141,279–50,143,398	Actin-depolymerizing factor 2	
				Jr07_36200	108986297	50,144,101–50,146,776	Pentatricopeptide repeat-containing protein At2g39620	
				Jr07_36210	108986366	50,148,093–50,152,954	Elongator complex protein 1	
				Jr07_36250, Jr07_36260, Jr07_36270	109015291	50,169,570–50,172,850	THO complex subunit 4B	
				Jr07_36280	108986386	50,171,698–50,172,477	THO complex subunit 4A	
				Jr07_36290, Jr07_36300, Jr07_36310	108986360	50,174,720–50,195,227	**Uncharacterized protein**	
AX-171017658	1	10,234,077	10,227,829–10,244,488	No gene	–	–	–	
**Kernel filling ratio**								
AX-171048523	10	19,688,911	19,686,439–19,688,911	No gene	–	–	–	
AX-171583500	3	3,603,319	3,596,654–3,609,718	Jr03_04860	108980538	3,597,955–3,599,758	Alpha-1,3-arabinosyltransferase XAT3	
				Jr03_04870, Jr03_04880	109014312	3,606,572–3,611,958	Transcription initiation factor IIB-2	
AX-171540343	13	6,539,102	6,523,102–6,557,694	Jr13_09170	108993396	6,521,880–6,523,699	Probable aquaporin PIP2-8	
				Jr13_09180	108993404	6,525,283–6,528,372	Pre-mRNA-splicing factor SYF2	
				Jr13_09220	108993314	6,543,826–6,546,850	Blue copper protein	
				Jr13_09240	108993378	6,552,307–6,558,488	Probable ubiquitin-conjugating enzyme E2 23	
AX-171117250	9	12,230,952	12,208,938–12,255,984	Jr09_03370	108982558	12,251,655–12,255,887	Serine/threonine-protein kinase ATM	
**Weight related traits**								
**Nut weight**								
AX-171175345	8	26,618,252	26,611,559–26,618,252	Jr08_19090	108985800	26,608,414–26,613,583	Phosphoenolpyruvate carboxykinase (ATP)	
				Jr08_19100	108985801	26,615,257–26,618,938	**Uncharacterized protein**	
AX-170573680	3	7,355,283	7,338,541–7,356,913	Jr03_09310	109003589	7,339,392–7,342,382	14 kDa zinc-binding protein	
				Jr03_09320, Jr03_09330	109003588	7,343,193–7,344,784	Sulfiredoxin, chloroplastic/mitochondrial	
				Jr03_09340	109003587	7,345,868–7,351,659	Phosphoglycerate kinase 3, cytosolic	
				Jr03_09350, Jr03_09360, Jr03_09370	109003586	7,353,891–7,362,252	**Chaperonin 60 subunit alpha 2, chloroplastic**	
AX-171083810	6	31,463,066	31,364,479–31,464,703	Jr06_15230, Jr06_15240	108985243	31,384,208–31,426,380	myb-related protein 308	
AX-170973887	12	3,304,218	3,296,379–3,352,059	Jr12_02800	108998258	3,304,480–3,341,673	Peroxisome biogenesis protein 1	
				Jr12_02810	108998259	3,345,122–3,359,362	FHA domain-containing protein FHA2	
AX-170605550	1	15,926,539	15,898,312–15,966,124	Jr01_17520	109011118	15,897,845–15,907,090	ER lumen protein-retaining receptor	
				Jr01_17530	109006016	15,940,330–15,943,514	Protein XRI1	
				Jr01_17540	109011117	15,947,614–15,951,582	Protein SICKLE	
				Jr01_17550, Jr01_17560	109006017	15,954,073–15,961,294	Purple acid phosphatase 15	
**X3 extreme groups**								
AX-170690867	14	1,248,953	1,248,139–1,248,953	Jr14_01880	109012316	1,248,515–1,262,435	**Beta-galactosidase**	LOI3
AX-170834489	1	19,207,870	19,202,146–19,218,695	No gene	–	–	–	
AX-170723157	10	4,802,938	4,802,821–4,802,938	No gene	–	–	–	
**Kernel weight**								
AX-171207844	1	39,963,556	39,934,883–39,963,556	Jr01_29160, Jr01_29170, Jr01_29180	109003484	39,947,452–39,948,585	Retrovirus-related Pol polyprotein from transposon RE1	
AX-171547969	7	3,283,684	3,279,632–3,290,086	Jr07_03110	108995343	3,280,892–3,285,736	**BEL1-like homeodomain protein 2**	
AX-170806411	4	23,487,069	23,484,729–23,487,069	No gene	–	–	–	
**Breaking yield**								
AX-171005810	14	26,821,528	26,821,528–26,851,219	No gene	–	–	–	
AX-170746651	16	17,458,649	No block	No gene	–	–	–	
**Cracking related traits**								
**Nut face strength**								
AX-170722428	11	13,511,848	13,503,419–13,528,275	Jr11_09630	109000368	13,524,918–13,540,197	Putative GPI-anchor transamidase	
AX-170865366	2	32,096,896	32,095,993–32,104,213	Jr02_19150, Jr02_19160, Jr02_19170	109010833	32,094,342–32,100,753	**Protein TPX2**	
**Nut suture strength**								
AX-170748526	5	13,024,522	13,017,020–13,109,943	Jr05_10970	108986888	13,045,730–13,046,379	Lamin-like protein	
				Jr05_10980	108986890	13,046,665–13,056,464	Protein argonaute 4A	
AX-170908256	11	12,909,083	12,884,235–12,916,294	No gene	–	–	–	
**Shell Thickness**								
AX-170865411	2	32,128,087	32,126,341–32,131,231	Jr02_19210	109010875	32,127,046–32,130,960	**TVP38/TMEM64 family membrane protein slr0305**	
**Nutritional components**								
**Monounsaturated fatty acids**								
AX-170701328	14	26,730,713	26,728,860–26,731,202	No gene	–	–	–	
AX-171494826	9	21,100,618	No block	No gene	–	–	–	
AX-170973656	12	3,155,205	3,139,539–3,155,205	Jr12_02680	108998216	3,151,847–3,153,051	Putative clathrin assembly protein At4g40080	
AX-170996807	14	21,159,561	No block	No gene	–	–	–	
AX-171536319	7	47,712,322	No block	Jr07_32830	108992563	47,710,331–47,712,379	**Calmodulin**	
**Tocopherols**								
AX-171134134	10	34,010,861	34,010,496–34,015,586	Jr10_22690	–	34,011,505–34,043,955	Receptor-like protein 22	LOI11
				Jr10_22700	108983893	34,012,093–34,014,327	Receptor-like protein 7	
AX-170893393	9	15,280,292	15,271,076–15,281,282	Jr09_05900	108994350	15,271,151–15,280,248	E3 ubiquitin-protein ligase HOS1	LOI12
				Jr09_05910	–	15,279,302–15,280,386	**Uncharacterized protein**	
				Jr09_05920	108994314	15,280,768–15,284,023	2-oxoglutarate-Fe(II) type oxidoreductase hxnY	
AX-170775823	13	23,062,305	23,062,229–23,062,659	No gene	–	–	–	
AX-170615496	8	29,149,403	29,145,942–29,153,435	No gene	–	–	–	
AX-171592220	5	16,978,410	16,976,697–16,991,665	Jr05_12430, Jr05_12440, Jr05_12450	108983737	16,978,531–16,980,684	Glucan endo-1,3-beta-glucosidase 14	
AX-170909834	7	3,266,820	3,265,033–3,267,053	No gene	–	–	–	
AX-171595297	14	7,508,539	7,508,539–7,508,805	No gene	–	–	–	
**Vitamin E activity**								
AX-171134134	10	34,010,861	34,010,496–34,015,586	Jr10_22690	–	34,011,505–34,043,955	Receptor-like protein 22	LOI11
				Jr10_22700	108983893	34,012,093–34,014,327	Receptor-like protein 7	
AX-170893393	9	15,280,292	15,271,076–15,281,282	Jr09_05900	108994350	15,271,151–15,280,248	E3 ubiquitin-protein ligase HOS1	LOI12
				Jr09_05910	–	15,279,302–15,280,386	**Uncharacterized protein**	
				Jr09_05920	108994314	15,280,768–15,284,023	2-oxoglutarate-Fe(II) type oxidoreductase hxnY	

*^*a*^Chr, Chromosome. ^*b*^Physical position given in bp. ^*c*^LD blocks are defined using the “solid spine of LD” method. ^*d*^The candidate genes in bold overlap the physical position of the associated SNP. ^*e*^LOI, locus of interest associated to several traits referred to a unique MTA or to several MTAs with close physical position.*

We identified a total of 75 different candidate genes within all the LD blocks (Table 3), with 13 candidate genes including some of the MTAs. Regarding nut morphometrics, shape, volume, and weight related traits, a gene coding for a *beta-galactosidase* was found to be associated with nut face diameter, nut volume, kernel volume, and the “x3 extreme groups.” We found a *receptor-like cytosolic serine/threonine-protein kinase RBK1* encoding gene for nut length, and an *L-Ala-D/L-amino acid epimerase* encoding gene associated with nut surface area and nut volume. We also found a *probable E3 ubiquitin-protein ligase ARI1* encoding gene linked with the nut Feret shape 3D, and a *protein TIFY 4B* encoding gene linked with empty space volume. Then, a *chaperonin 60 subunit alpha 2, chloroplastic* and a *BEL1-like homeodomain protein 4* encoding genes were found to be associated, respectively, with nut weight and kernel weight. In addition, for the cracking related traits, we determined a gene coding for a *protein TPX2-like* being involved in nut face strength, and a *TVP38/TMEM64 family membrane protein* encoding gene linked with the shell thickness. Finally, we found a candidate gene linked with the monounsaturated fatty acids content such as a *calmodulin* encoding gene. Interestingly, the SNP associated with this trait fell within an exonic sequence of this *calmodulin* encoding gene.

## Discussion

### The INRAE Walnut Germplasm Collection Has a High Degree of Fruit Phenotypic Variability

The walnut genetic resources of INRAE form an *ex situ* collection near Bordeaux, France, and gather numerous accessions of different geographical origin (Bernard et al., 2018a). By comparison with other walnut panels used for GWAS, we observed a larger degree of fruit phenotypic variability. For instance, in the panel used in Iran, the range for nut weight was between 7.71 and 20.11 g (Arab et al., 2019), whereas in our panel, combining all the years studied, the range was between 5.22 and 22.51 g. Similarly, in the work on suture strength reported by the University of California, Davis, the use of a texture analyzer to phenotype suture strength showed a variation between 6.94 to 63.14 kg-force (i.e., between 68.06 to 619.19 N) among the 556 accessions studied (mainly from 39 biparental progenies) corresponding to a variation of 551.13 N (Sideli et al., 2020). In our walnut germplasm, with only 170 accessions and combining all the years, the variation extends from 74.40 to 776.97 N. With the high phenotypic variability and the diversity of the INRAE walnut panel used for GWAS analysis, knowing that most of these traits are highly quantitative, we may expect to find major loci involved in their variation.

### MTAs of Correlated Traits Are Not Impacted by Different Phenotyping Methods

In our study, we attempted to improve the accuracy of phenotyping procedures for several traits across the following phenotyping years. This is the case for three of the nut morphometrics related traits: in 2017, we used an electronic caliper to measure nut length, nut face diameter, and nut profile diameter, but we decided to develop an X-ray CT method for the nuts phenotyped in 2018. We had some doubts concerning the impact of changing our phenotyping method on the GWAS results. However, the resulting BLUPs were reliable since we found a significant association on Chr14 for nut face diameter, which was also detected for nut volume and kernel volume, only measured in 2018 and for which no BLUPs were calculated. In addition, the three traits were significantly positively correlated.

Nut face diameter, nut volume, and kernel volume were all correlated traits and the MTA AX-170690867 on Chr14 in the LOI3 is associated with all of them (minor allele G: frequency 0.30, and major allele T: frequency 0.70), with major allele having a positive effect (+0.74 BLUPs unit, +2,101.08 mm^3^ and + 521.01 mm^3^, respectively). The trait “x3 extreme groups” was associated with the MTA AX-171170293 on Chr14 also in the LOI3 (minor allele A: frequency 0.47, and major allele G: frequency 0.53), with major allele having a negative effect (−9.83 BLUPs unit). The LOI3 including the two MTAs (AX-170690867 and AX-171170293) and two additional SNPs, was a haplotype block with five haplotypes, of which the one of interest had a frequency of only 0.23. The two MTAs were in moderate LD (*r*^2^ = 0.37) and belonged to the sequence of a *beta-galactosidase* encoding gene. This finding suggests that almost equivalent traits might be considered as a simple “repetition” of the phenotyping, leading preferentially to the detection of the major locus.

Although nut size is a complex trait, the association on Chr14 controlled three different dimensions of nut (nut face diameter, nut volume, and kernel volume), suggesting its crucial role in determining nut size. The explained phenotypic variance (R^2^) at this locus was high for each of the three traits (i.e., for nut face diameter: 26.36 vs. 19.80, 5.88, and 3.22). However, by phenotyping correlated traits, we were able to detect minor and more specific loci involved in nut size. For instance, if nut volume and kernel volume are sharing the major association on Chr14, we found other specific minor associations. These findings have implications for selection: if it is possible to select superior genotypes for bigger nut size in general (MTA on Chr14), it remain possible for instance, to select for bigger kernel volume using only minor loci.

### Comparison of MTAs Detected in Other Germplasm Collections for Similar Traits

For cracking related traits, using different phenotyping methods and FarmCPU model, the MTA AX-170748528 (Chr5, 13,023,760 bp) was found to be associated with the suture strength (Sideli et al., 2020). This MTA was only 762 bp apart from the SNP just below the significant threshold obtained for the suture strength initial rupture. These two SNPs are located in the same region close to a gene coding for a *lamin-like protein*. Similarly, three MTAs were identified on the Chr11 using FarmCPU model, depending on the phenotype recorded with texture analyzer (Sideli et al., 2020). The genomic region spans from 10,995,387 bp to 13,714,234 bp. We found one suggestive association signal within this interval (at 12,909,083 bp). The walnut germplasm collections of INRAE and University of California, Davis, share numerous accessions, such as “Chandler,” “Robert Livermore,” “Vina,” and diverse representing accessions from China, Japan, and France. The two germplasm collections are also related, since many parents of common California varieties are French (Marrano et al., 2019a). The shared genetic background can explain why we found the same major loci. However, our GWAS panel was smaller compared to Sideli et al. (2020), and this could be the reason we did not obtain significant marker association with this trait.

On the contrary, we did not find any association in common with the association study performed on nut traits by Arab et al. (2019). This study was based on a panel of 95 genotypes representing local populations across four different Iranian areas from valleys to mountains. The panel represented a large part of Iranian walnut populations’ genetic diversity that is not represented in our panel, explaining why we found different results. This means that our markers can be used at University of California, Davis, for the selection of superior genotypes, but they will be less efficient at the University of Tehran, and vice versa. This finding proves the difficulty to develop markers for selection on a global scale (Mohammadi et al., 2020), and that it will be important to create a large and more diverse collection of walnut accessions for future work, representing the genetic diversity across the world.

### Contribution to Better Understanding of the Genetic Control of Fruit Quality

Nut size results from a complex signaling pathway involving cell division and expansion. Serine/threonine kinases are enzymes catalyzing the phosphorylation from ATP to an amino acid residue (Diallo and Prigent, 2011). Several studies suggested that receptor-like cytosolic kinases contribute to plant development, particularly in cell wall function (Muto et al., 2004; Steinwand and Kieber, 2010) and cell morphogenesis (Becraft, 2002). Similarly, beta-galactosidases are enzymes catalyzing hydrolyzation of galactosyl residues of hemicellulose and pectin from the cell wall (Smith and Gross, 2000; Yang et al., 2018), and L-Ala-D/L-amino acid epimerases are enzymes catalyzing the epimerization of various dipeptides (Lukk et al., 2012), involved in the peptidoglycan pathway, essential for cell wall integrity in bacteria, but also in land plants, although lacking in those peptidoglycans (Yang et al., 2013). The *beta-galactosidase*, *L-Ala-D/L-amino acid epimerase-like*, and *receptor-like cytosolic serine/threonine-protein kinase RBK1* are therefore interesting candidate genes involved in nut morphometrics.

Along with nut size, kernel weight and the easiness of cracking are important traits for walnut quality. We found a *BEL1-like homeodomain protein 4* encoding gene as involved in kernel weight. In *Arabidopsis thaliana*, BEL1 protein is a commonly found transcription factor required for ovule morphogenesis (Modrusan et al., 1994), whereas in potato, BEL1-type proteins enhance tuberization (Sharma et al., 2014). Therefore, BEL1 is likely involved in determining kernel weight in walnut. In addition, we found a *protein TPX2-like* encoding candidate gene for nut face strength. TPX2 is a protein phosphorylated during mitosis interphase acting as a spindle assembly factor for microtubules in the nucleus, and is therefore crucial for cell division.

Using the “solid spine of LD” for the definition of the LD blocks, we highlighted complementary candidate genes for several MTAs. Nut volume is the trait with the highest number of candidate genes found. In addition to the gene coding for a *beta-galactosidase* related to the most significant association on Chr14, we found an *expansin-A4* and a *scarecrow-like protein 14* encoding genes on Chr7, involved in loosening of cell wall during growth (Cosgrove, 2000) and flower bud transition (Quan et al., 2019). We also found a *calcium-binding protein PBP1* encoding gene on Chr9, supporting the central role of calcium during fruit growth when Ca^2+^ uptake increases (Song et al., 2018). As Sideli et al. (2020), we identified a gene encoding a *lamin-like protein* for nut suture strength. Lamins are proteins part of the lamina, providing a mechanical support to the nuclear envelope and also acting on nucleus size and shape (Ciska and Moreno Díaz de la Espina, 2014).

The selection of appropriate model and threshold levels is crucial in GWAS. Among the two models implemented, FarmCPU provided the highest number of significant associations. FarmCPU has a higher detection power than previous GWAS models since it controls for false positives while reducing false negatives; it uses a MLMM divided into fixed effect and random effect model iteratively (Liu et al., 2016). FarmCPU has not been widely used for complex traits in crops because of a lacking comparison with previous existing models. However, MLMM and FarmCPU models have been recently compared using well-known GWAS dataset of soybean and maize, and the authors confirm FarmCPU’s ability to outperform previous GWAS models (Kaler et al., 2020). In addition, the authors claim that MLMM did not find any significant marker since it uses a too conservative multiple comparison adjustment method. For this reason, we decided to consider also signals below the 1% Bonferroni threshold to avoid false negatives.

### First List of Candidate Genes Involved in Fatty Acids and Vitamin E Contents Variation in Kernel

For the first time in walnut, we discovered SNPs and candidate genes associated with monounsaturated fatty acids and tocopherols contents. Primary metabolites in plants such as lipids and vitamins are synthesized by numerous multi-enzymatic complexes, and we found that the significant SNP on Chr7 associated with monounsaturated fatty acids content falls within a gene coding for a *calmodulin*. In *de novo* fatty acids synthesis, the fatty acid synthase requires NAD(P)H as cofactor, and the dependent nicotinamide adenine dinucleotide (NAD) signaling is thought to be highly regulated by calmodulin (Tai et al., 2019). However, additional phenotyping data are necessary to confirm the MTAs and the candidate genes identified.

## Conclusion

By combining a highly accurate characterization of crucial traits for walnut quality with a high diverse walnut germplasm collection, a dense SNP genotyping, and newly available multi-locus models, we identified numerous MTAs, particularly for traits related to nut size. We confirmed major loci involved in suture strength, and we proposed candidate genes for fruit quality, mainly linked to cell wall function and calcium signaling. After phenological traits, the INRAE walnut germplasm collection proved its suitability for GWAS. It will make easy to select genitors allowing future release of new walnut cultivars meeting the criteria required by both consumers and producers.

## Data Availability Statement

The datasets presented in this study can be found in online repositories. The names of the repository/repositories and accession number(s) can be found below: https://doi.org/10.15454/XPKII8, Portail Data INRAE.

## Author Contributions

AB performed all the genetic analyzes and wrote the manuscript. JC assisted AB in performing, analyzing, and interpreting the data of weight and cracking related traits. AD and AM provided their experience in GWAS to interpret the results. ED, FL, and AB conceived and coordinated the research. All authors revised the manuscript.

## Conflict of Interest

The authors declare that the research was conducted in the absence of any commercial or financial relationships that could be construed as a potential conflict of interest.

## Footnotes

1 www.fao.org/faostat/

2 https://hardwoodgenomics.org/bio_data/2539069?tripal_pane=group_downloads

## Supplementary Material

Supplementary Figure 1 Histograms of the 25 studied traits for each year of evaluation for the walnut GWAS analysis.

Supplementary Figure 2 Manhattan plots of traits related to fruit shape, cracking and nutritional components studied in the walnut GWAS analysis. For each trait, MLMM Manhattan plot and Q-Q plot are shown at the top, and FarmCPU Manhattan plot and Q-Q plot are shown at the bottom. Horizontal green line corresponds to 1% Bonferroni threshold automatically implemented in GAPIT.

Supplementary Table 1 Description of the 25 studied traits with the methodologies used for the different years of evaluation, for the walnut GWAS analysis.

Supplementary Table 2 Phenotypic raw dataset of the 25 traits studied in the walnut GWAS analysis.
