# Supplementary figures and images for: Genome-Wide Association Study Reveals Candidate Genes Involved in Fruit Trait Variation in Persian Walnut (Juglans regia L.)
Source: Front Plant Sci. 2021 Jan 8;11:607213. doi: 10.3389/fpls.2020.607213 (PMC7873874; doi:10.3389/fpls.2020.607213)

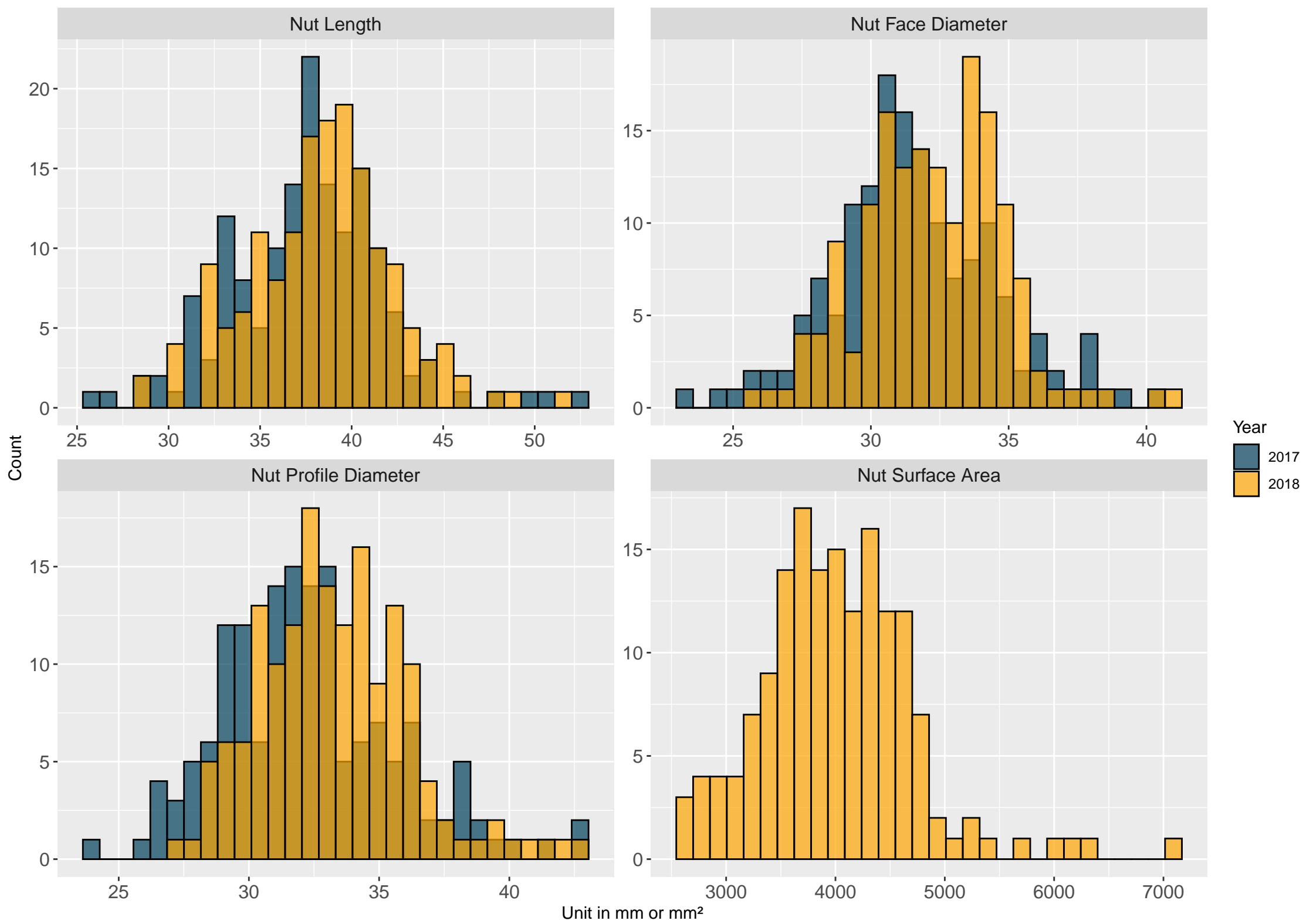

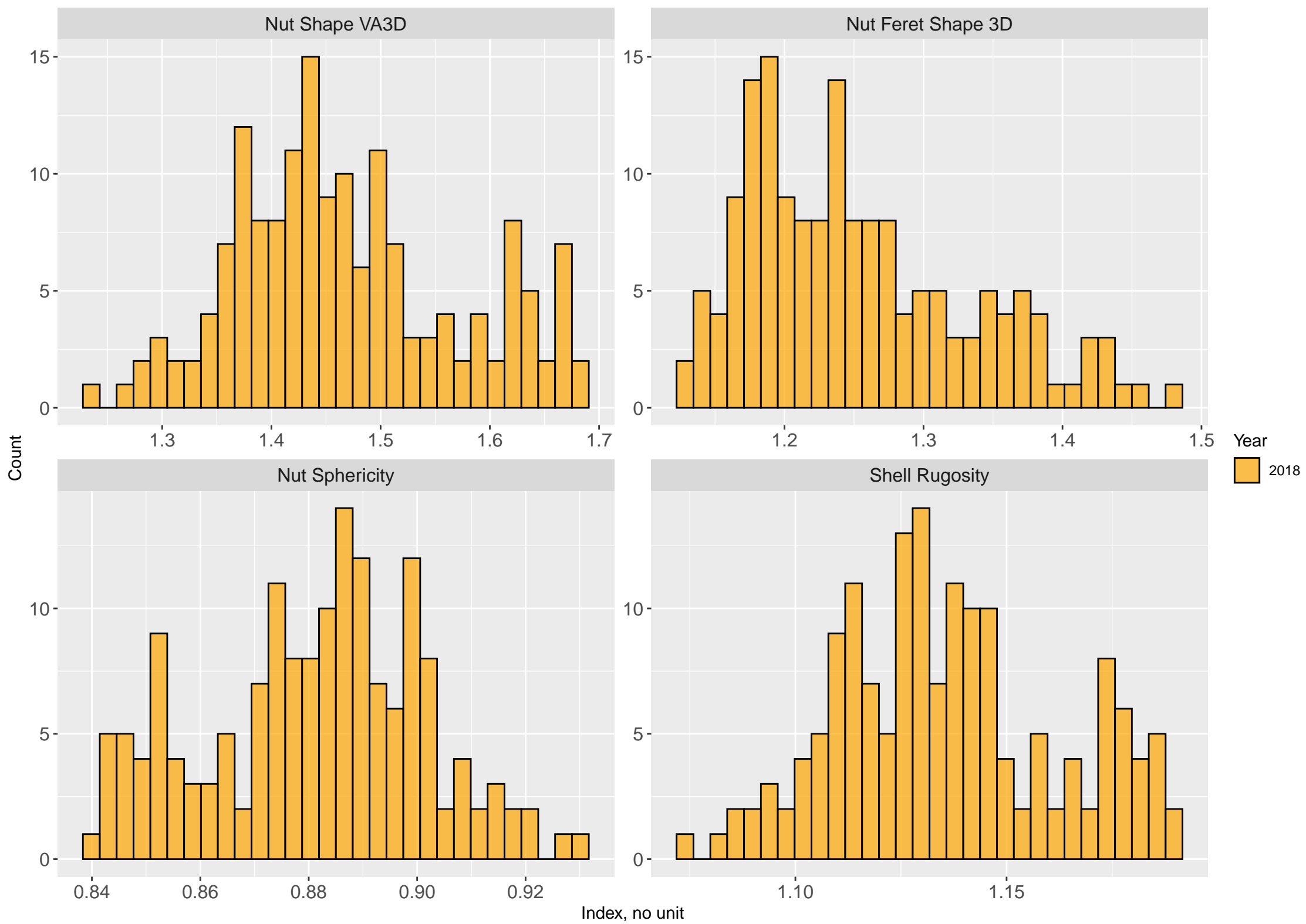

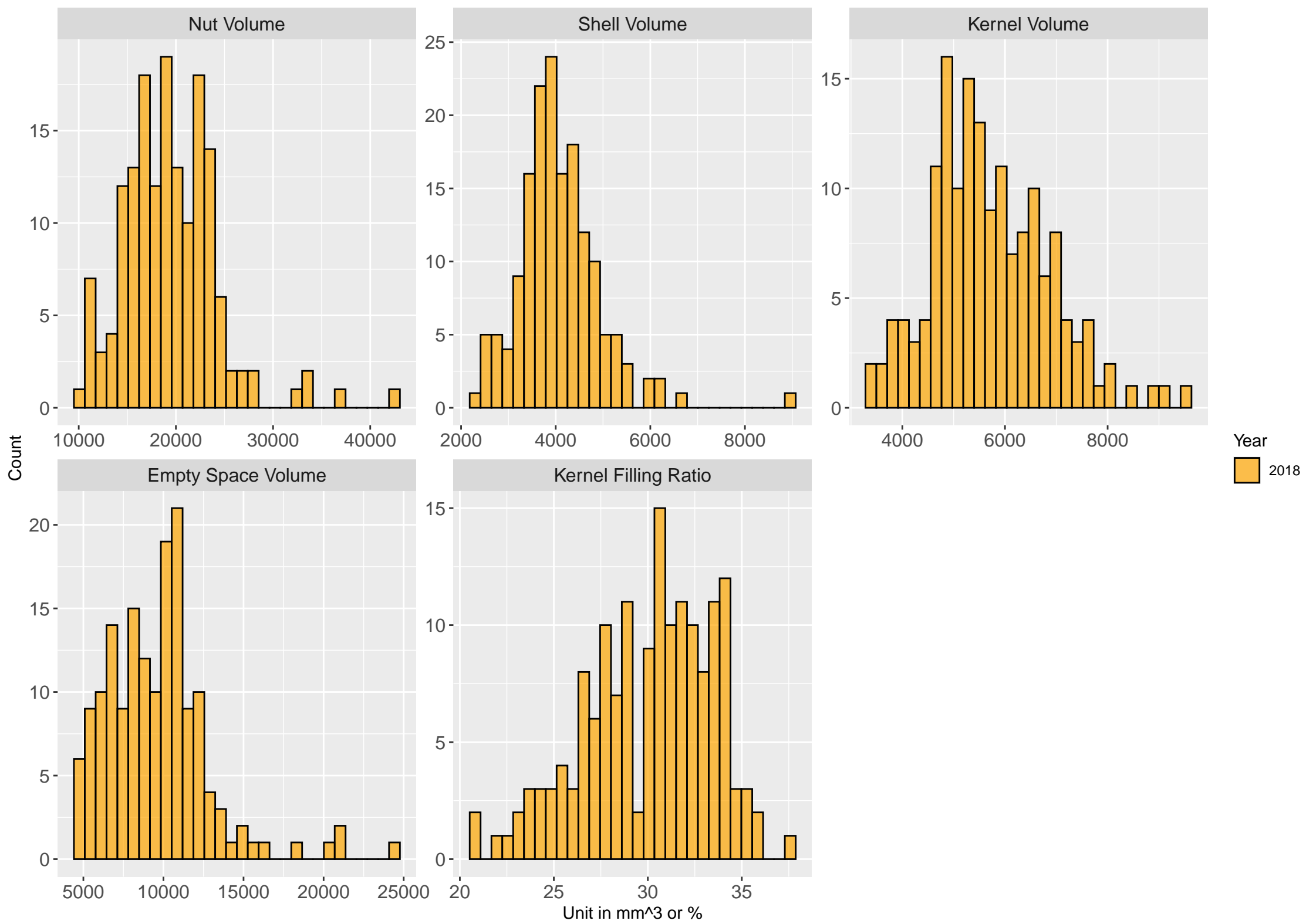

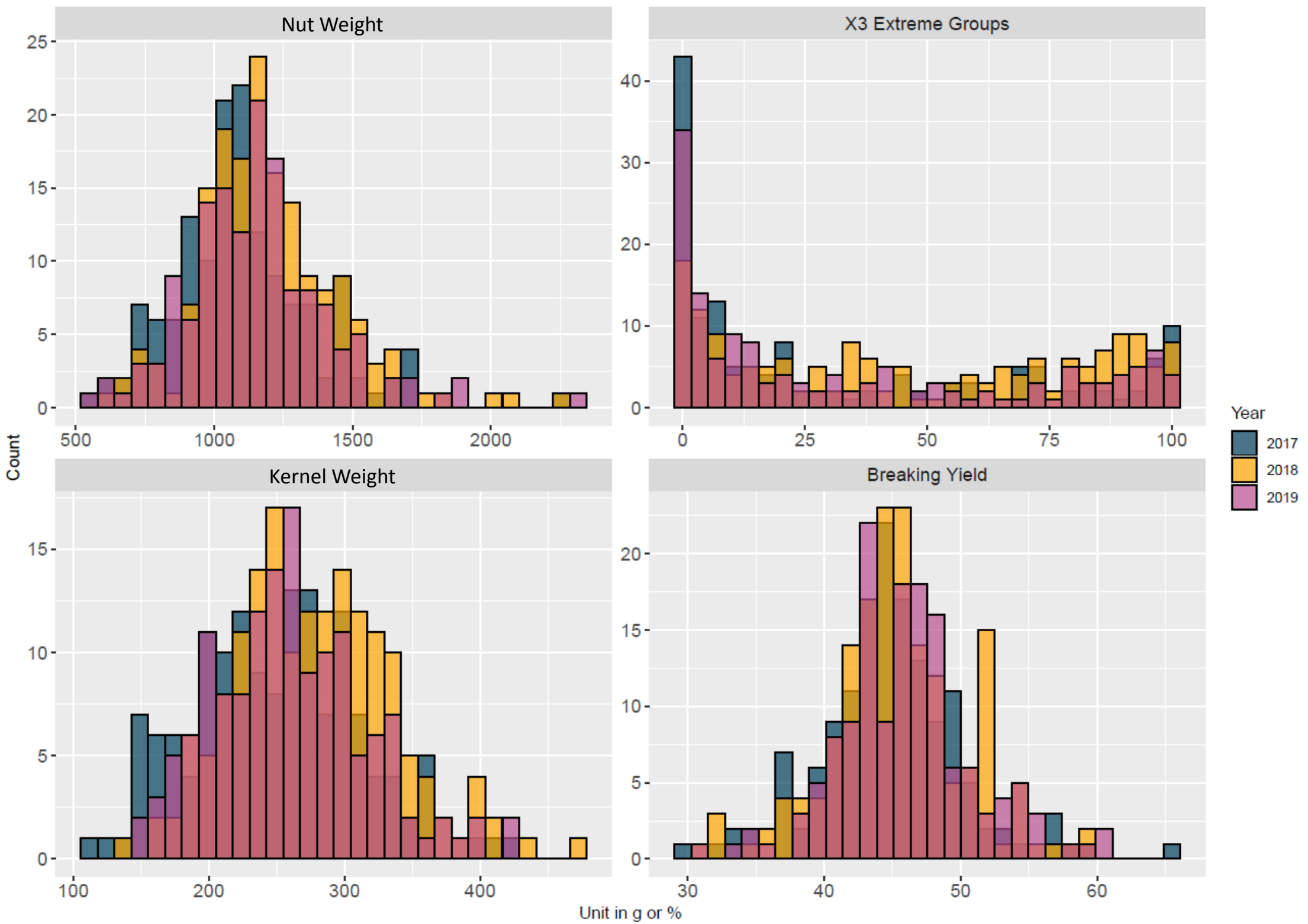

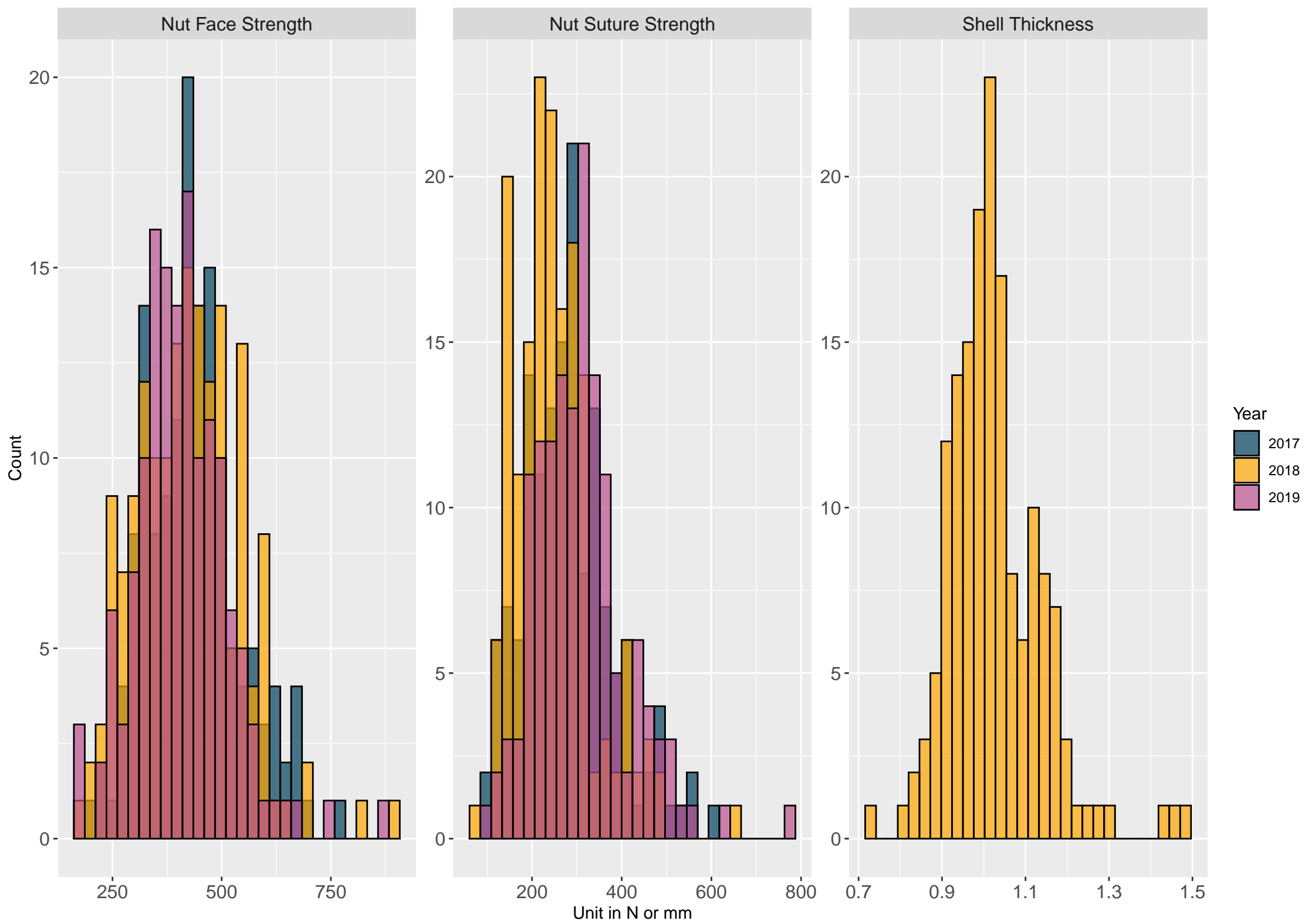

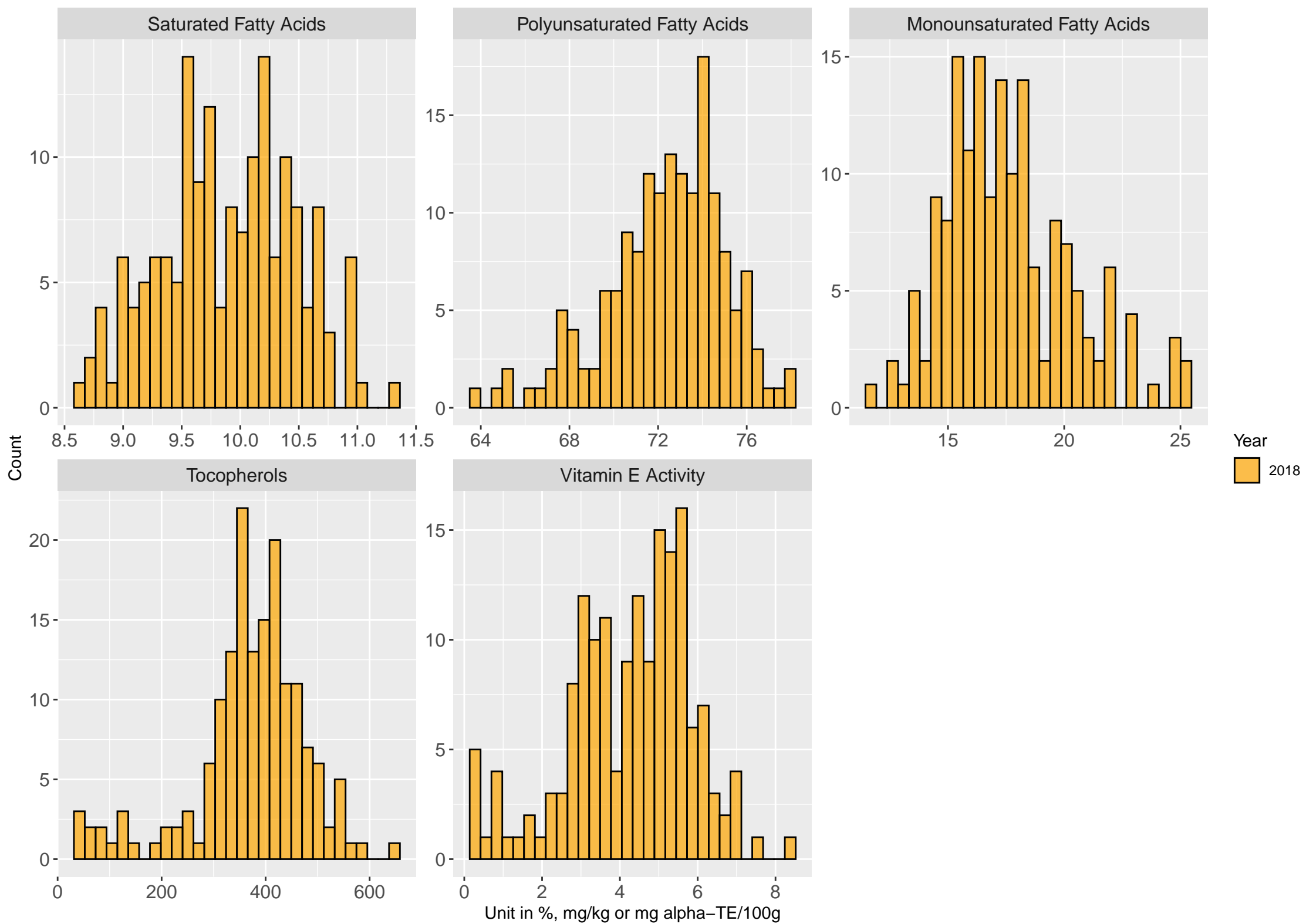

Supplement: Supplementary Figure 1 — Histograms of the 25 studied traits for each year of evaluation for the walnut GWAS analysis. [file Data_Sheet_1.PDF]
