# Supplementary material for: Genome-Wide Association Study Reveals Candidate Genes Involved in Fruit Trait Variation in Persian Walnut (Juglans regia L.)
Source: Front Plant Sci. 2021 Jan 8;11:607213. doi: 10.3389/fpls.2020.607213 (PMC7873874; doi:10.3389/fpls.2020.607213)

Nut Shape VA3D, MLMM (top) & FarmCPU (bottom), 2018

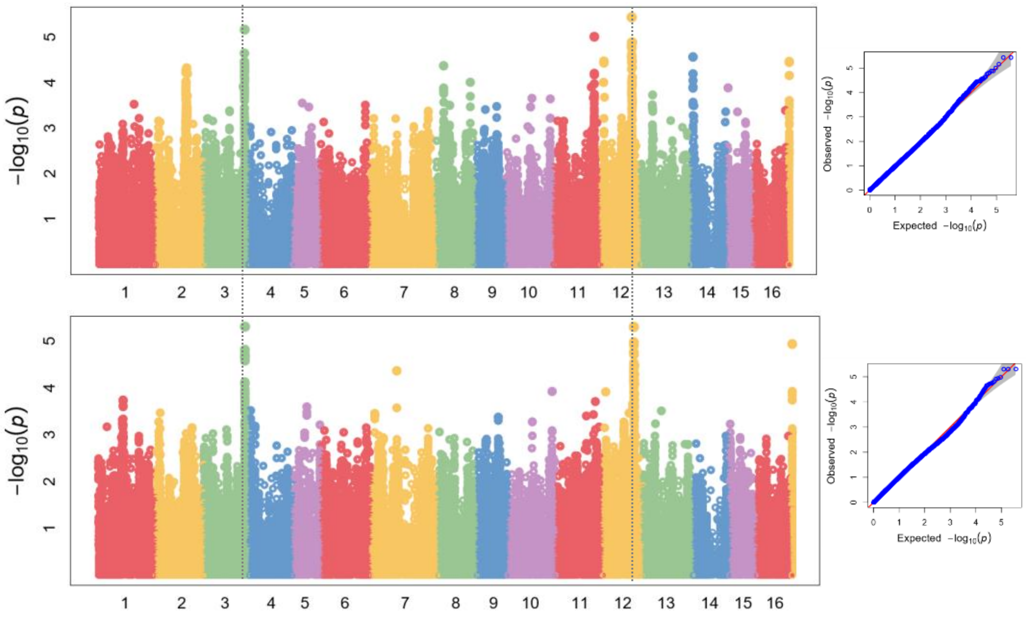

Nut Feret Shape 3D, MLMM (top) & FarmCPU (bottom), 2018

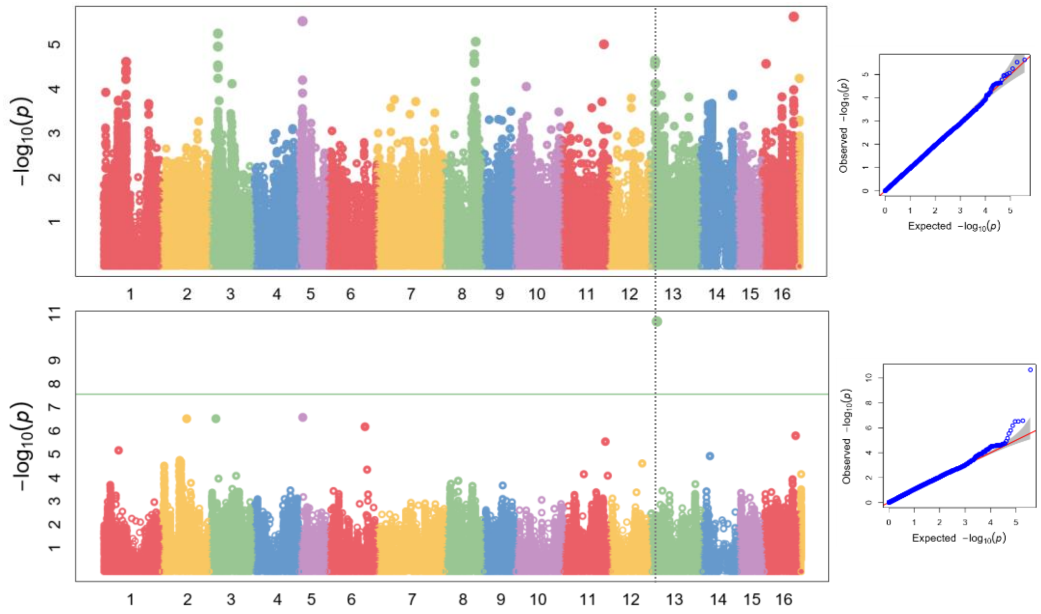

Nut Sphericity, MLMM (top) & FarmCPU (bottom), 2018

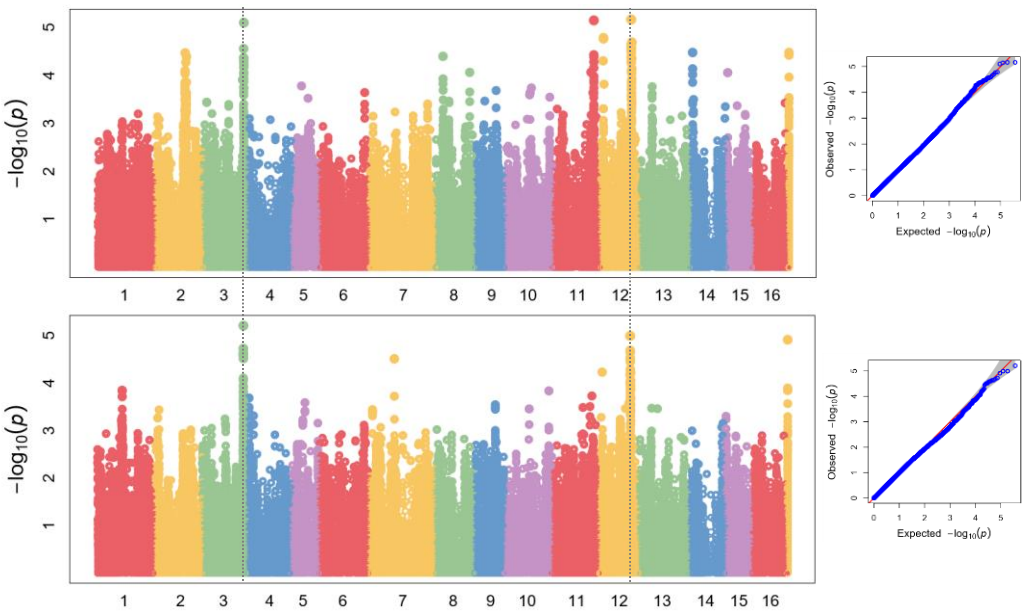

Shell Rugosity, MLMM (top) & FarmCPU (bottom), 2018

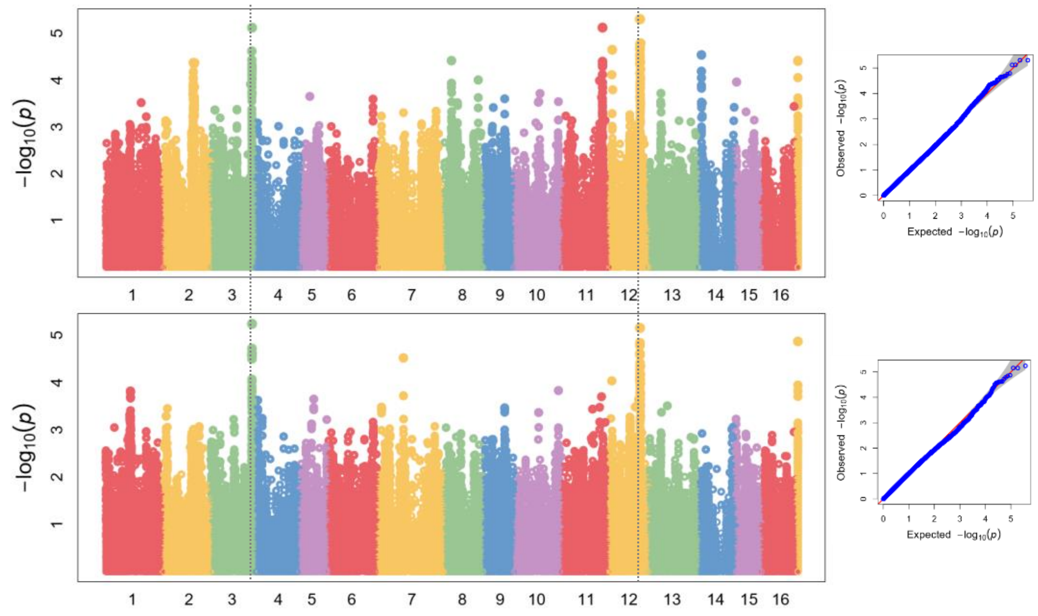

Nut Face Strength, MLMM (top) & FarmCPU (bottom), BLUPs 2017/2018/2019

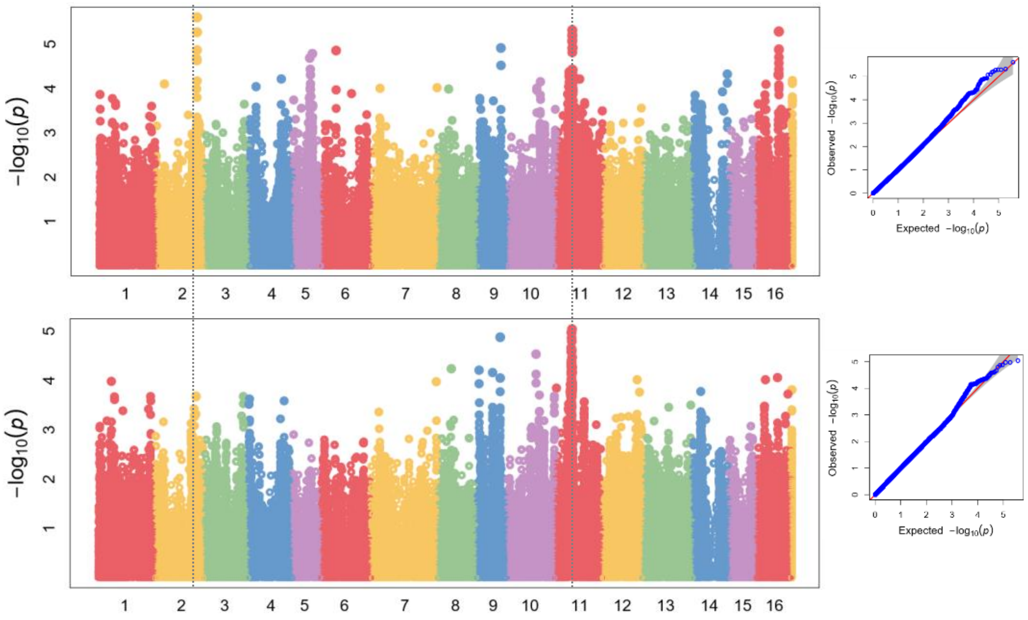

Nut Suture Strength, MLMM (top) & FarmCPU (bottom), BLUPs 2017/2018/2019

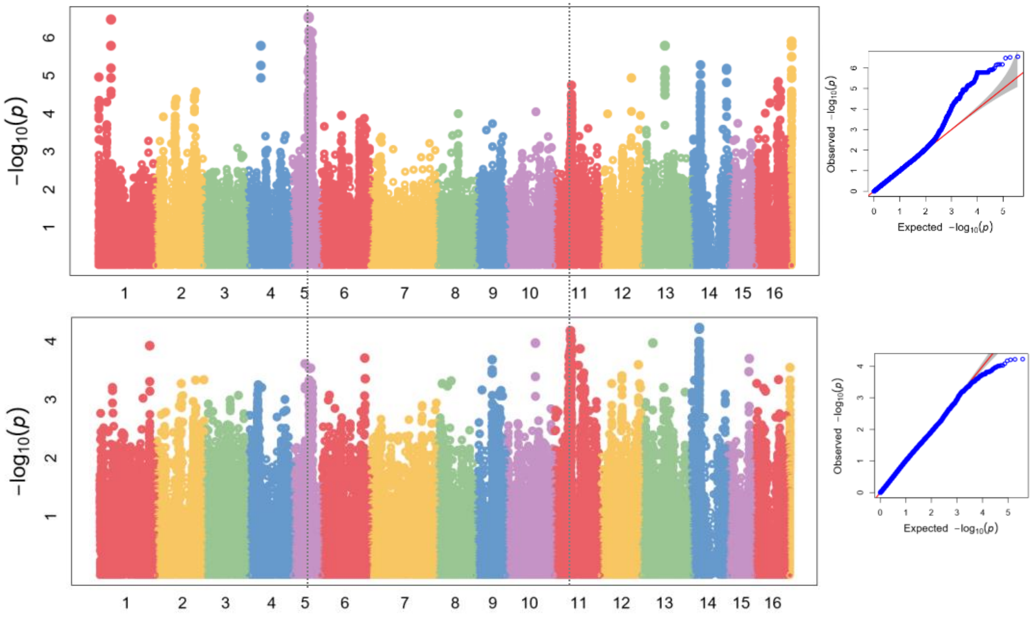

Shell Thickness, MLMM (top) & FarmCPU (bottom), 2018

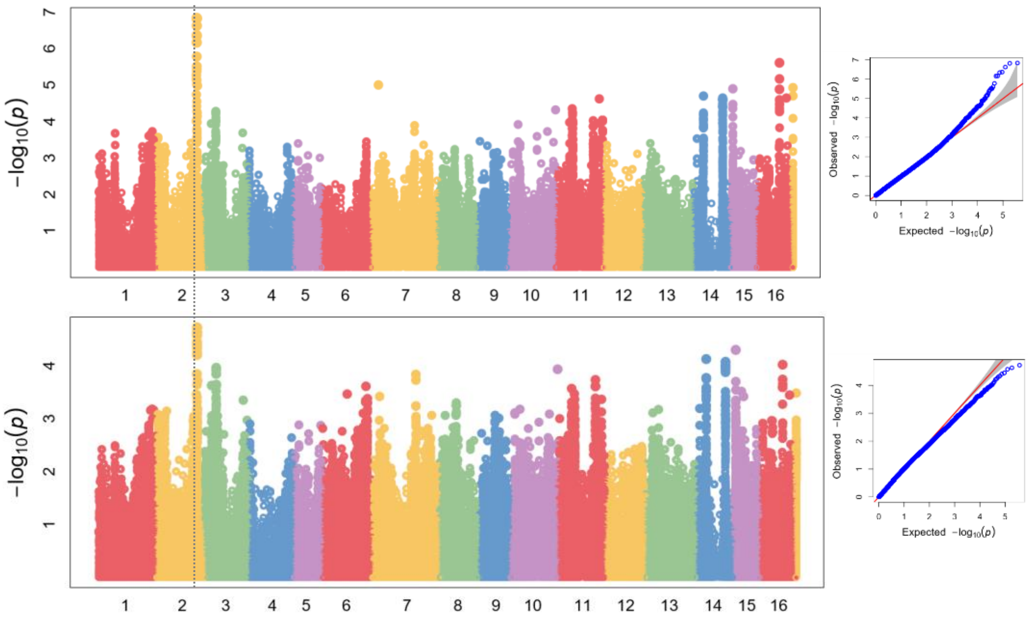

Saturated FA, MLMM (top) & FarmCPU (bottom), 2018

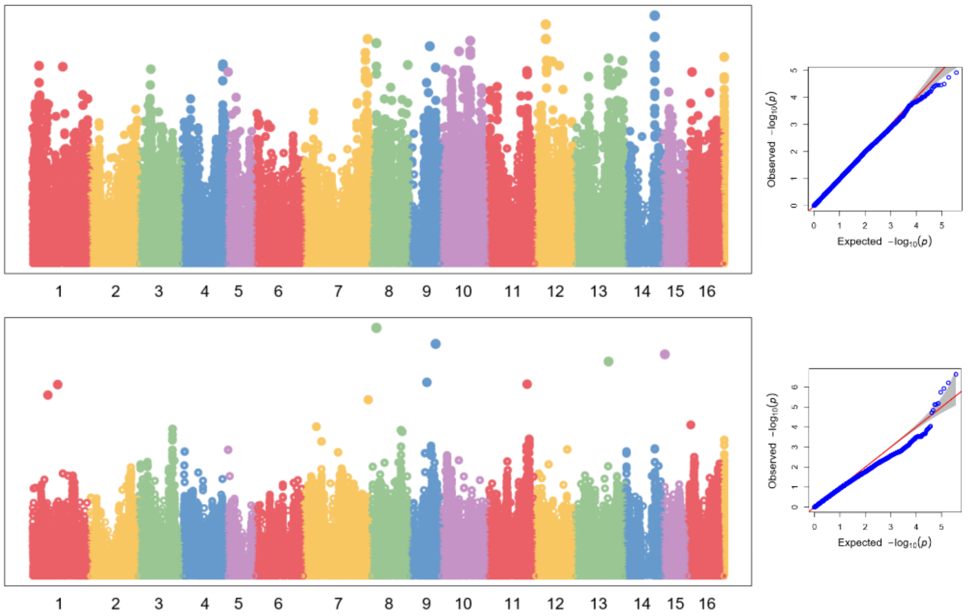

Polyunsat. FA, MLMM (top) & FarmCPU (bottom), 2018

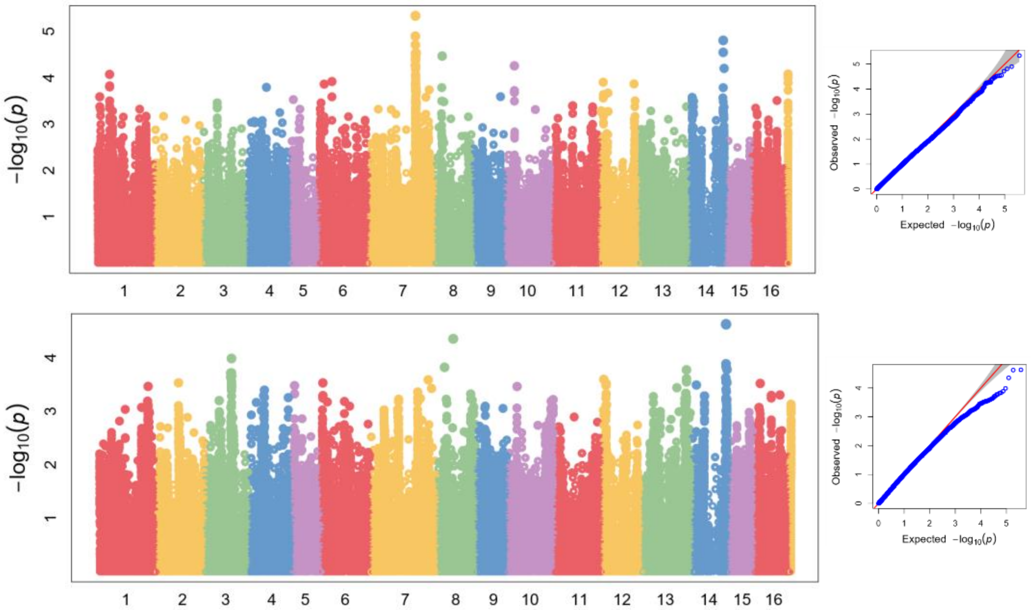

Monounsat. FA, MLMM (top) & FarmCPU (bottom), 2018

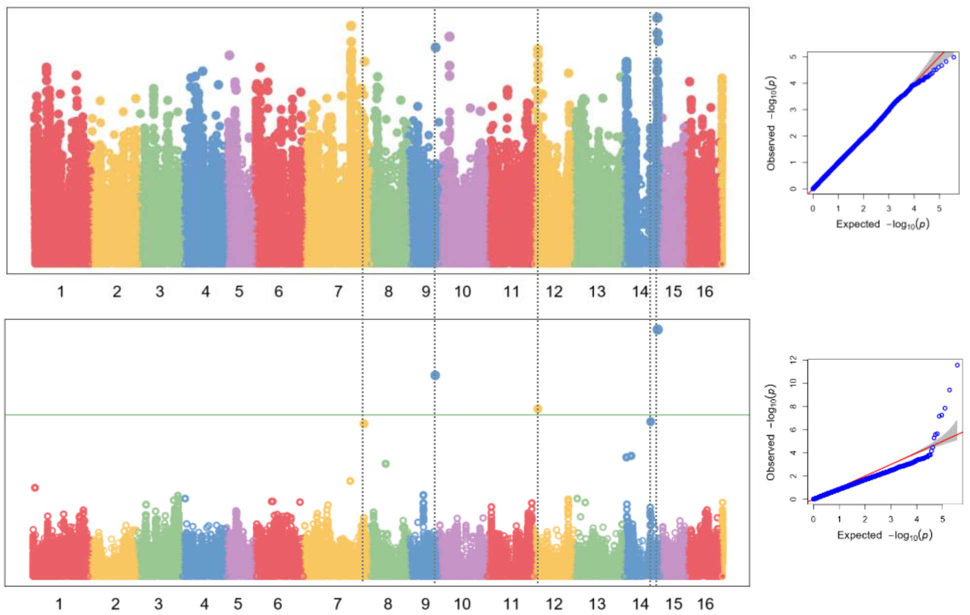

Tocopherols, MLMM (top) & FarmCPU (bottom), 2018

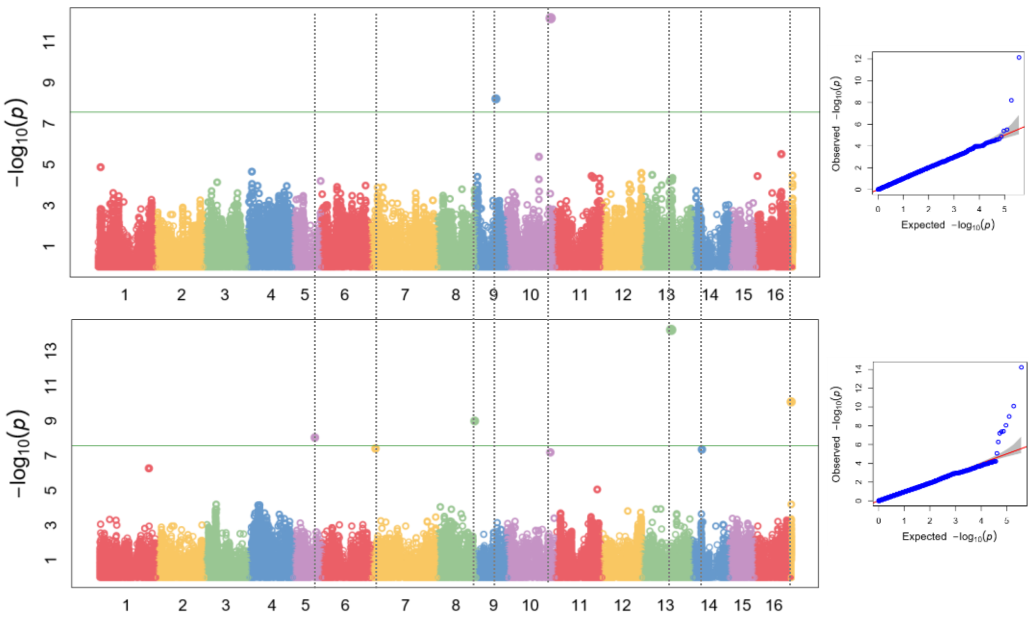

Vit. E Activity, MLMM (top) & FarmCPU (bottom), 2018

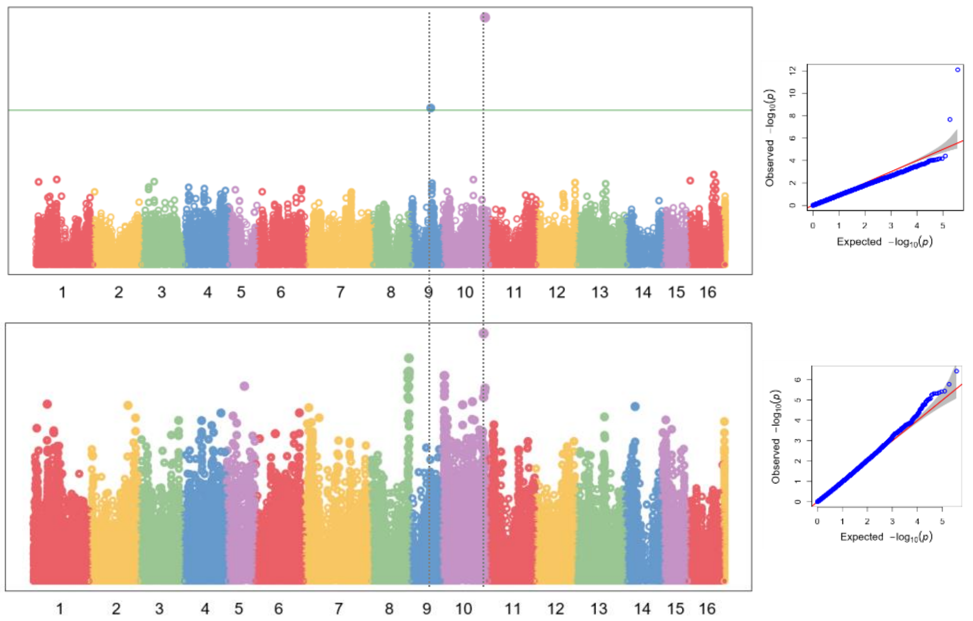

Supplement: Supplementary Figure 2 — Manhattan plots of traits related to fruit shape, cracking and nutritional components studied in the walnut GWAS analysis. For each trait, MLMM Manhattan plot and Q-Q plot are shown at the top, and FarmCPU Manhattan plot and Q-Q plot are shown at the bottom. Horizontal green line corresponds to 1% Bonferroni threshold automatically implemented in GAPIT. [file Data_Sheet_2.PDF]
